# Multipulse sodium magnetic resonance imaging for multicompartment quantification: Proof-of-concept

**DOI:** 10.1038/s41598-017-17582-w

**Published:** 2017-12-12

**Authors:** Alina Gilles, Armin M. Nagel, Guillaume Madelin

**Affiliations:** 1 Center for Biomedical Imaging, Department of Radiology, New York University School of Medicine, New York, NY 10016 USA; 2 Institute of Radiology, University Hospital Erlangen, Friedrich-Alexander-University Erlangen-Nuremberg, 91054 Erlangen, Germany

## Abstract

We present a feasibility study of sodium quantification in a multicompartment model of the brain using sodium (^23^Na) magnetic resonance imaging. The proposed method is based on a multipulse sequence acquisition and simulation at 7 T, which allows to differentiate the ^23^Na signals emanating from three compartments in human brain *in vivo*: intracellular (compartment 1), extracellular (compartment 2), and cerebrospinal fluid (compartment 3). The intracellular sodium concentration *C*_1_ and the volume fractions *α*_1_, *α*_2_, and *α*_3_ of all respective three brain compartments can be estimated. Simulations of the sodium spin 3/2 dynamics during a 15-pulse sequence were used to optimize the acquisition sequence by minimizing the correlation between the signal evolutions from the three compartments. The method was first tested on a three-compartment phantom as proof-of-concept. Average values of the ^23^Na quantifications in four healthy volunteer brains were *α*_1_ = 0.54 ± 0.01, *α*_2_ = 0.23 ± 0.01, *α*_3_ = 1.03 ± 0.01, and *C*_1_ = 23 ± 3 mM, which are comparable to the expected physiological values ${{\boldsymbol{\alpha }}}_{{\bf{1}}}^{{\boldsymbol{theory}}}$ ∼ 0.6, ${{\boldsymbol{\alpha }}}_{{\bf{2}}}^{{\boldsymbol{theory}}}$ ∼ 0.2, ${{\boldsymbol{\alpha }}}_{{\bf{3}}}^{{\boldsymbol{theory}}}$ ∼ 1, and ${{\boldsymbol{C}}}_{{\bf{1}}}^{{\boldsymbol{theory}}}$ ∼ 10–30 mM. The proposed method may allow a quantitative assessment of the metabolic role of sodium ions in cellular processes and their malfunctions in brain *in vivo*.

## Introduction

Magnetic resonance imaging (MRI) is an important clinical diagnostic tool usually based on the detection of protons (^1^H) in tissues. Protons yield the strongest nuclear magnetic resonance (NMR) signal in human tissue. Along with displaying the morphology and a wide range of contrasts, the visualization of tissue functions becomes increasingly important. The sodium cations (^23^Na^+^) play a major role in many fundamental physiological functions in human tissues^1^. The level of low intra- and higher extracellular ^23^Na concentrations ($\sim$10–30 mM and $\sim$140 mM, respectively)^1–10^ is tightly regulated in healthy mammalian tissue. Many transmembrane ion transporters, most importantly the Na^+^/K^+^-ATPase (sodium-potassium pump), maintain this concentration gradient and regulate membrane depolarization as well as cell volume, intracellular pH and transepithelial transport. Dysregulation of the Na^+^/K^+^-ATPase can provoke an increase of intracellular sodium concentration. Alterations in the distribution of sodium cations across the cell membrane may therefore be indicative of pathological conditions, either due to disease or during cell damage induced by therapy. Brain tumor studies^11–13^ have shown an increase of the total ^23^Na signal in lesions, likely due to dysregulations of the Na^+^/K^+^-ATPase. This change in total sodium signal can originate from changes in intracellular sodium concentration or variations in extracellular volume fraction (with constant extracellular sodium concentration of 140 mM). It is therefore highly desirable to develop imaging methods to obtain information about the modification of sodium distributions in different cellular spaces in human tissue under pathological conditions *in vivo* in order to understand where these sodium signal changes come from.

Sodium MRI is challenging, as the resulting images generally suffer from low signal-to-noise ratio (SNR). The relative NMR receptivity^14^ of the ^23^Na nuclei is approximately 9.27% of the proton (^1^H) NMR receptivity. In addition, the sodium signal originates from a low concentration of Na^+^ ions in brain tissues *in vivo*, compared to ^1^H concentration: the average ^23^Na concentration is in the range 30–50 mM, compared to approximately 0.8 × 110 M = 88 M average ^1^H concentration from water that composes about 80% of the brain volume. Altogether, the ^23^Na MRI signal is approximately 21,000 times lower than the ^1^H signal *in vivo*. The SNR of sodium MRI can be increased by acquiring the data at ultra-high magnetic fields (≥3 T) and low resolutions (in the order of 5 mm isotropic). Moreover, due to its spin 3/2, the sodium nucleus exhibits a quadrupolar moment that strongly interacts with the electric field gradients of its environment, generating short relaxation times T_1_ and biexponential T_2_, in the order of sub-milliseconds to tens of milliseconds. Short T_1_ can be used to increase the number of averages using short repetition times (TR), while short T_2_ will implicate the need of using ultrashort echo time (UTE) sequences for sodium data acquisition.

Most of ^23^Na MRI studies investigate the total ^23^Na signal and therefore lack specificity about the origin of sodium signal variations and their link to useful metabolic information. Ideally, the intracellular and extracellular sodium signals can selectively be acquired using chemical shift reagents, but only in animal studies^15^ as these reagents can’t be used in humans due to their toxicity. Non-invasive relaxation-based approaches, such as inversion recovery (IR)^16,17^, which suppress signal components with long longitudinal relaxation times such as cerebrospinal fluid (CSF), can also be implemented to increase the sensitivity of the method to intracellular sodium concentration changes^18,19^. Triple-quantum filtering (TQF) techniques^20,21^ which can separate the signals from ^23^Na^+^ ions with different restricted mobility can also be implemented to achieve the same goal, but generally face low SNR problems. In general, both IR (which is based on T_1_ relaxation) and TQF (based on T_2_ relaxation) methods assumed that only the sodium ions in the intracellular space are bound or restricted in their mobility, due to strong interaction with other metabolites and organelles in that space. However, there is also evidence that sodium ions in extracellular space can interact with many molecules, membranes and metabolites in their environment, and generate residual signal after IR or TQF filtering^22^, that can be mismatched with the intracellular signal.

**Figure 1:**
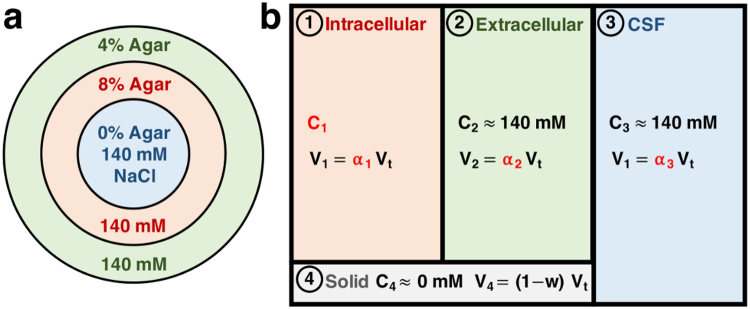
Multicompartment models of agar gel phantom and brain. (**a**) Cylindrical phantom with 140 mM sodium chloride (NaCl) in variably concentrated concentrically disposed agar gel compartments. In cross section: 0% agar in the center, 8% agar in the inner ring, and 4% agar in the outer ring. Sodium ions in the 8%, 4%, and 0% agar gel compartments are considered to show similar relaxation characteristics to ^23^Na in intracellular, extracellular, and CSF compartments in brain, respectively (matching colors). (**b**) Four-compartment model for brain tissue. ^23^Na ions are present in the intracellular (1), extracellular (2) and CSF (3) compartments of the human brain. Sodium signal from the solid compartment (4) is negligible. Notations are, for *j* = 1 to 4: *C*_*j*_ = sodium concentrations, *V*_*j*_ = volumes, *α*_*j*_ = volume fractions, *w* = water fraction. Assumptions for brain model: *w* = *α*_1_ + *α*_2_ + *α*_3_, with *w* = 0.8, total volume *V*_*t*_ = *V*_1_ + *V*_2_ + *V*_3_ + *V*_4_, and *C*_2_ = *C*_3_ = 140 mM. Unknown values of interest are in red: *C*_1_, *α*_1_, *α*_2_, and *α*_3_.

The quantitative multicompartment-multipulse ^23^Na MRI method presented in this study can be seen as one new approach to investigate intra- and extracellular sodium *in vivo* which is based on both the T_1_ and T_2_ relaxation of the different compartments. Using a multipulse sequence acquisition and a quantification method based on sodium spin 3/2 dynamics simulation during this sequence (with 15 pulses in our case), we can selectively separate the ^23^Na signal from three different compartments in human brain tissue *in vivo*. The compartments differ in their relaxation times and are assumed to correspond to the intracellular (IC), the extracellular (EC), and the cerebrospinal fluid (CSF) spaces, which were assigned the numbers 1, 2, and 3, respectively, in our study (see Fig. 1). An additional solid compartment (assigned number 4), was also included in our tissue model, where no sodium ions are present. This solid compartment does not generate sodium signal but needs to be taken into account to calculate the volume fractions in brain. The signal separation with a multipulse acquisition allows a quantitative non-invasive estimation of IC sodium concentration (*C*_1_), as well as the volume fractions *α*_1_ (IC), *α*_2_ (EC) and *α*_3_ (CSF) of the different compartments at 7 T. According to the literature^1–10,23–28^, in healthy brain tissues (parenchyma), *C*_1_ is in the range 10–30 mM, *α*_1_ is in the range 0.5–0.6, *α*_2_ is in the range 0.2–0.3, and ${\alpha }_{3}\sim 1$ in CSF and $\sim 0$ elsewhere. The volume fraction of the solid compartment is assumed to be in the range 0.2–0.3, calculated as 1 minus the water fraction in parenchyma, which is in the range 0.7–0.8^29,30^. The sum of all non-solid volume fractions (*α*_1_, *α*_2_, *α*_3_) should therefore be equal to the water fraction *w* in brain tissue. In this preliminary model, the vascular space, which occupies around 3% of brain volume^31^, was assumed to be negligible and part of the extracellular compartment. The EC compartment therefore includes the interstitial and vascular spaces.

Measuring all these values (C1, *α*_1_, *α*_2_ and *α*_3_) and their conspicuous changes could help identifying pathologies at an early stage. Changes in intra- and extracellular volume fractions can result from fluid effusion, increase of vascularization, or disruption of cells for example^24^, and provide information about edema or tumor angiogenesis^25,32^. The intracellular sodium concentration on the other hand could be used as a biomarker of neurodegeneration and loss of cell viability in stroke^33^, in multiple sclerosis^34^, or in Alzheimer’s disease^35^. It can also prove useful for assessing tumor malignancy^36,37^ or to monitor cancer therapy^16,38^. In this pilot study, we present a proof-of-concept of the multipulse quantification method on a three-compartment phantom with known sodium concentration and volume fractions (Fig. 1a), followed by its application to brain *in vivo* on four volunteers using a four-compartment brain tissue model (Fig. 1b). The steps for data acquisition and quantification are described in detail in the Methods section. The proposed multicompartment-multipulse ^23^Na MRI method could pave the way for the visualization *in vivo* of pathologies originating from metabolic dysfunction at the cellular level, such as loss of cell viability or cell volume, and variations in cell packing.

## Results

### Sodium signal acquisitions and simulations in phantom and brain

**Figure 2:**
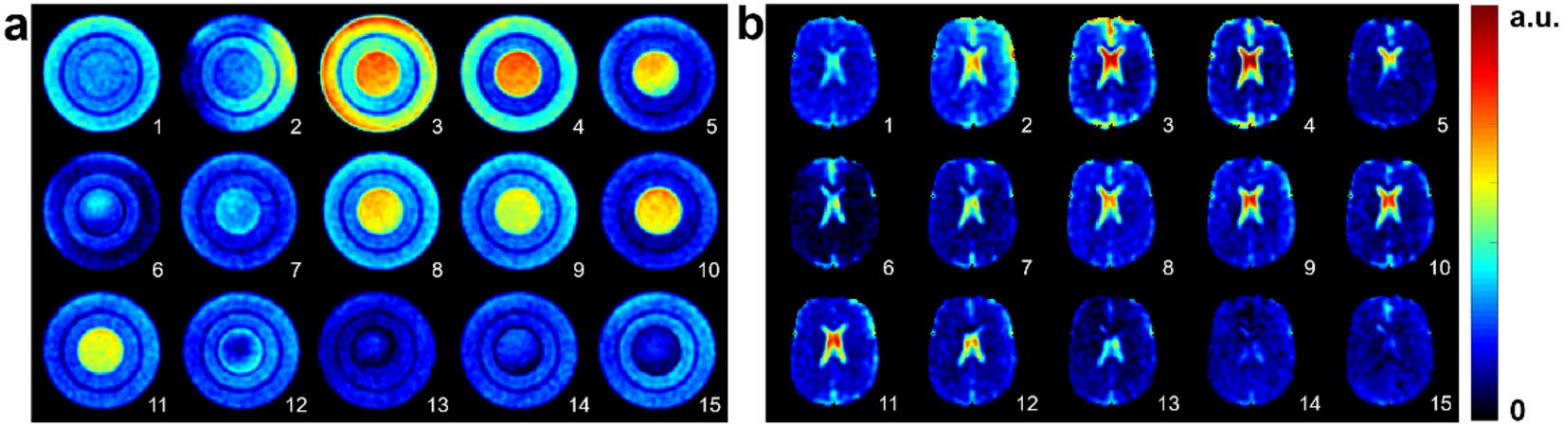
Axial sodium images acquired with 15-pulse FLORET sequence. Sequence parameters were: flip angles *θ*_*i*_ = 16°, 150°, 54°, 16°, 43°, 105°, 49°, 56°, 120°, 65°, 64°, 114°, 44°, 44°, 119°, phases *ϕ*_*i*_ = 73°, 79°, 39°, 43°, 182°, 64°, 146°, 104°, 3°, 125°, 21°, 138°, 39°, 68°, 172°, delays *τ*_*i*_ = 5 ms after each pulse (*i* = 1 to 15). A extra delay was added after the last RF pulse for completion to the chosen TR. (**a**) Phantom with three compartments: center 0% agar gel, inner ring 8% agar gel, outer ring 4% agar gel, sodium concentration = 140 mM all compartments. (**b**) *In vivo* brain measurements in healthy volunteer.

Figure 2a shows an example of axial ^23^Na images of a three-compartment phantom (0%, 4% and 8% agar gel concentrations with 140 mM ^23^Na concentration) from a 15-pulse FLORET^39^ acquisition. Figure 2b shows axial ^23^Na images of the brain of a healthy volunteer using the same 15-pulse sequence.

**Figure 3:**
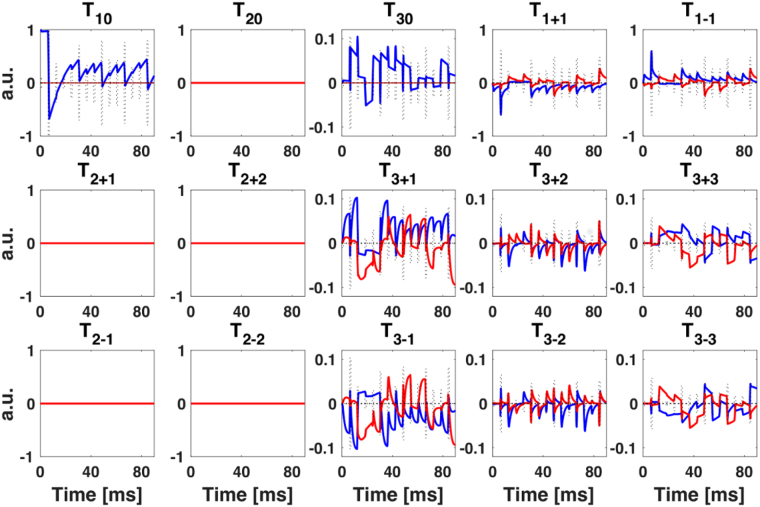
Time evolution of the irreducible spherical tensor operators (ISTOs) *T*_*lm*_: real (blue) and imaginary (red) part of *T*_*lm*_ for spin-3/2 sodium signal deriving from intracellular brain compartment as simulated for 15-pulse sequence applied in the present study. See Methods for sequence details. The entire set of 15 tensor operators and their developments over the sequence contribute to the acquired MR signal after each pulse, represented by the *T*_1±1_ operator. The dashed line represent the RF pulses (height = relative flip angle).

Figure 3 shows an example of the simulation of the evolution of the irreducible spherical tensor operators (ISTOs) representing the full spin dynamics of the sodium spin 3/2 in the intracellular compartment during the 15-pulse sequence. The evolution of all spherical tensors was simulated during and between RF pulses. The final acquired magnetization *T*_1±1_ – or equivalently *I*_x_ and *I*_y_ (see Methods) – depends on the evolution and relaxation of all single, double and triple quantum coherences (SQC, DQC, TQC). In this example, the DQCs did not evolve as they occur only when there is a residual anisotropic quadrupolar coupling between the spins 3/2 and their environment, due to strong anisotropy in the system. This effect is generally detected in solid samples, but rarely in biological soft tissues such as the brain^40^. However, due to the biexponential T_2_ relaxation of the sodium spins in the intracellular space (due to restricted motion and strong quadrupolar interaction with surrounding metabolites as main cause of relaxation), TQCs can evolve during the multipulse sequence, and will indeed affect the final magnetization *T*_1±1_ measured with the RF coil.

**Figure 4:**
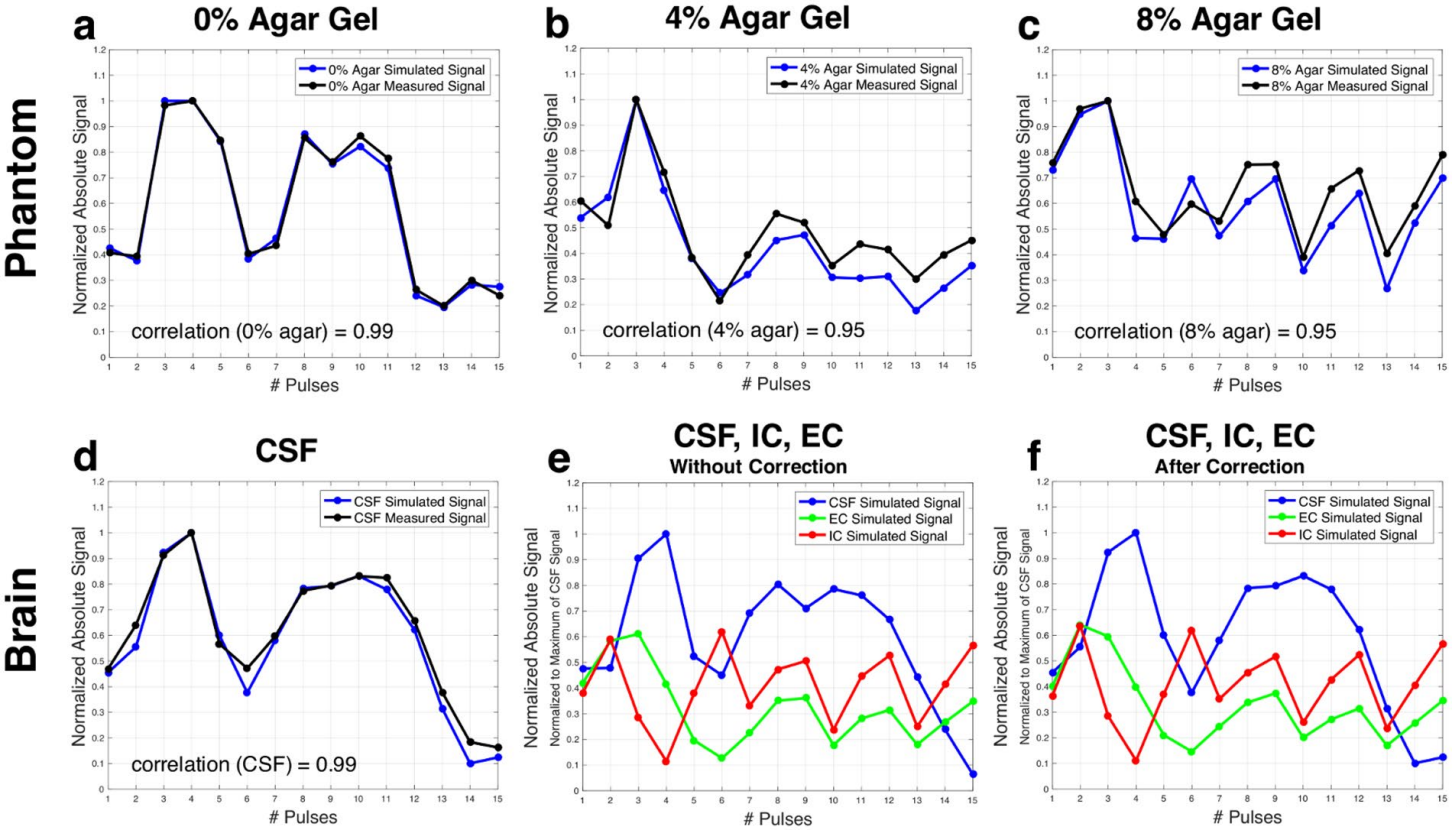
Time evolution of the maximum signal of different compartments in phantom and in brain after each RF pulse. (**a–d**) Comparison of simulated (blue) and measured (black) maximum magnitude ^23^Na signals after each pulse of the 15-pulse FLORET sequence for (**a**) 0% agar phantom compartment, showing a correlation of 0.99, (**b**) 4% agar phantom compartment, with a correlation of 0.95, (**c**) 8% agar phantom compartment with a correlation of 0.95, and (**d**) cerebrospinal fluid (CSF) in the brain, showing a correlation of 0.99. The 15-point signals were all normalized to the the maximum signal point in each compartment. (**e,f**) Absolute sodium signals over 15 pulses as simulated for three compartments in the brain: CSF, intracellular (IC), and extracellular (EC), before (**e**) and after (**f**) frequency offset and ${B}_{1}^{+}$ correction procedures. See Table 1 for corresponding correlation coefficients between these three signals.

Figure 4 shows the representative evolution of the maximum absolute sodium signal after each of the 15 pulses of the acquisition in the phantom and in brain. Figure 4a–d represents the comparisons of simulated (blue) and measured (black) normalized magnitude ^23^Na signal evolution after each of the 15 pulses of the sequence for the different phantom compartments (a: 0% agar, b: 4% agar, c: 8% agar) and (d) for CSF in the lateral ventricles in brain. The evolutions of the average signal intensities in black were measured in region of interest (ROI) measurements over three consecutive slices. The simulated signal evolutions in blue represent the evolution of maximum absolute magnitude transverse magnetization (*T*_1±1_) after each pulse, and results from simulations of ISTOs using different relaxation times for the different compartments, and applying frequency offset as well as ${B}_{1}^{+}$ correction factors (as described in Methods). Correlation coefficients as a measure of the agreement between measured and simulated data are 0.95 (for 4% and 8% agar) and 0.99 (for 0% agar and CSF). Figure 4e,f shows the normalized absolute ^23^Na signal evolutions after each of the 15 pulses as simulated for the three brain compartments (IC, EC, and CSF), (e) before and (f) after frequency offset and ${B}_{1}^{+}$ correction procedures.

Table 1 presents the correlation coefficients between signal evolutions from the different compartments in the phantom (0%, 4%, 8% agar) and in brain (IC, EC, CSF) as measured and simulated, before and after correction procedures. These correlation coefficients were minimized during the 15-pulse optimization procedure (see Methods for a detailed description of the correction and optimization procedures). The correlation between the ^23^Na signals measured in the 4% and 8% agar gel compartments was 0.7 (which is the most difficult to minimize due to close relaxation times of these two compartments), while it is 0.57 between 0% and 4%, and almost 0 between 0% and 8%. The simulated signal evolutions from different brain compartments show correlations of around −0.5 to 0.3. The simulated signal evolutions from IC and EC compartments in particular show correlations of around 0.1 (after correction).

**Table 1:** Correlation coefficients between signal evolutions during the multipulse acquisition. The correlation coefficients were calculated between signal evolutions from three different compartments in the phantom (0%, 4%, and 8% agar) and in brain (CSF, IC, and EC), as measured in ROIs in the phantom compartments (first column), as well as from signal simulations in both phantom and brain, before and after ${B}_{1}^{+}$ and frequency offset correction (second and third column, respectively). Correlation coefficients between 4% and 8% agar compartments signal developments are around 0.7. Simulated signal developments from IC and EC compartments in human brain tissue for the present 15-pulse sequence show correlations of around 0.1. *Abbreviations:* CSF, EC, and IC stand for cerebrospinal fluid, extracellular, and intracellular compartments in the brain.

	Measured	Correlation Simulated *Before Correction*	Simulated *After Correction*
**Phantom**			
0% and 4% Agar	0.55	0.41	0.57
0% and 8% Agar	0.05	0.01	0.04
4% and 8% Agar	0.67	0.77	0.70
**Brain**			
CSF and EC		0.23	0.32
CSF and IC		−0.52	−0.41
EC and IC		0.02	0.12

Table 2 lists the relaxation times used for the simulations. Average phantom relaxation times were measured using a relaxation time dictionary using a 20-pulse sequence (see Methods). Sodium relaxation times in brain compartments *in vivo* were taken from the literature^15,41–44^.

**Table 2:** Relaxation times. Monoexponential T_1_ and biexponential ${{\rm{T}}}_{2l}^{\ast }$ and ${{\rm{T}}}_{2s}^{\ast }$ relaxation times used for ^23^Na signal simulations for phantom (0%, 4%, and 8% agar) and brain (CSF, EC, and IC) compartments. Phantom relaxation times were determined using a relaxation time dictionary. Brain relaxation times were taken from the literature^15,41–44,48^.

	**T**_1_ (ms)	${{\rm{T}}}_{2l}^{\ast }$ (ms)	${{\rm{T}}}_{2s}^{\ast }$ (ms)
**Phantom**			
0% Agar	60	52	50
4% Agar	54	32	5
8% Agar	38	26	2
**Brain**			
CSF	64	56	56
EC	46	30	3.5
IC	24	14	2

The SNR values in all 15 phantom images were measured as: 26, 26, 44, 34, 23, 15, 20, 30, 29, 23, 26, 21, 13, 18, 21. The SNR values in all 15 whole brain images were measured as: 14, 20, 19, 16, 10, 8, 11, 15, 14, 12, 15, 12, 9, 10, 10.

### Sodium quantification in phantom

**Figure 5:**
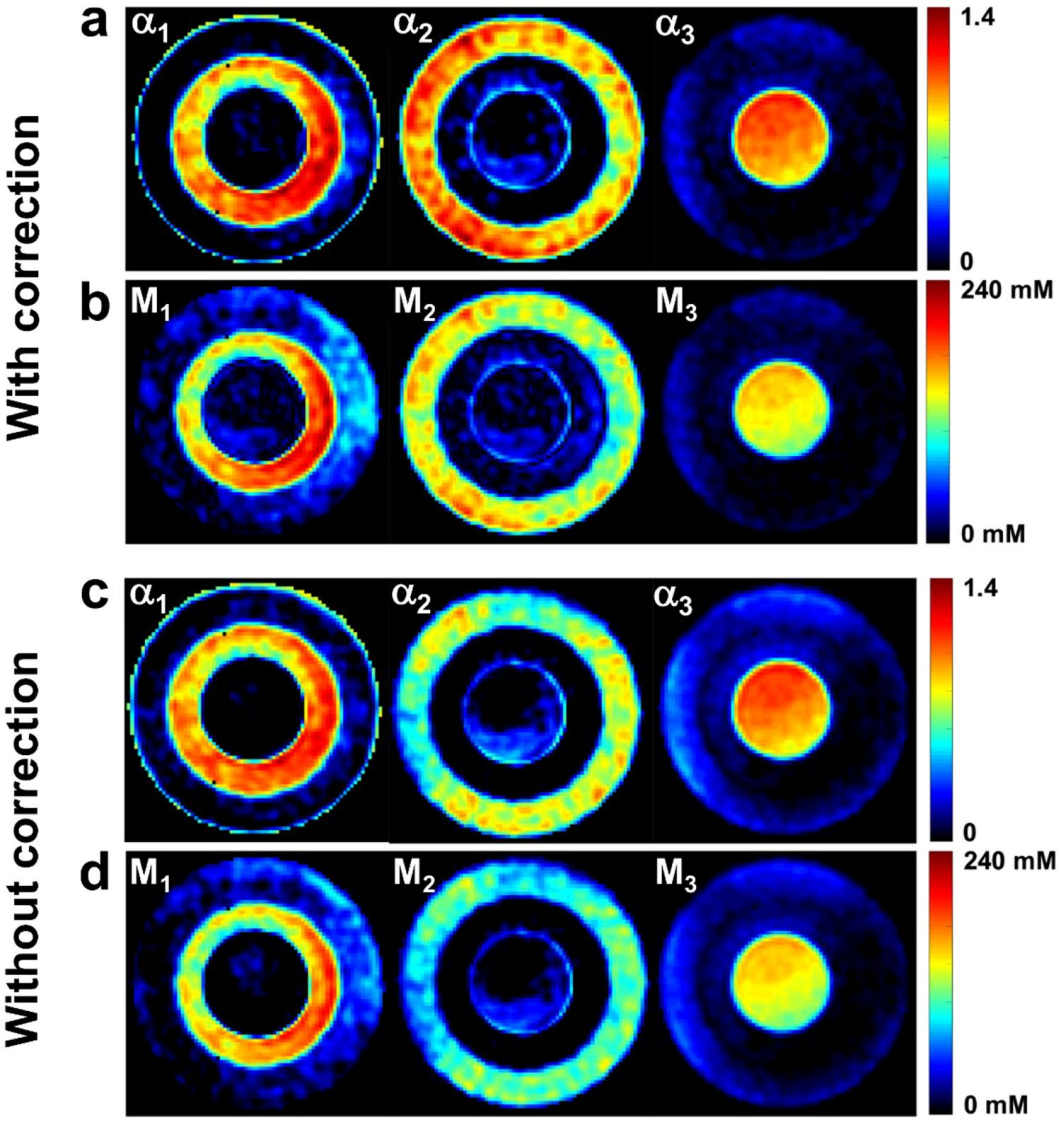
Example of sodium data quantification and filtering in phantom, with and without frequency offset and ${B}_{1}^{+}$ correction. (**a**) Volume fractions *α*_1_, *α*_2_. and *α*_3_ with correction. (**b**) Apparent total concentrations *M*_1_, *M*_2_, and *M*_3_ with correction. (**c**) Volume fractions *α*_1_, *α*_2_. and *α*_3_ without correction. (**d**) Apparent total concentrations *M*_1_, *M*_2_, and *M*_3_ without correction. Compartment-wise ROI evaluations generate mean values ± standard deviation of *α*_1_ = 0.82 ± 0.12, *α*_2_ = 0.99 ± 0.11, *α*_3_ = 1.01 ± 0.07, *M*_1_ = 129 ± 30 mM, *M*_2_ = 139 ± 15 mM, *M*_3_ = 140 ± 10 mM when frequency offset and ${B}_{1}^{+}$ correction was included in the quantification, and *α*_1_ = 0.97 ± 0.13, *α*_2_ = 0.70 ± 0.12, *α*_3_ = 0.99 ± 0.09, *M*_1_ = 143 ± 29 mM, *M*_2_ = 97 ± 16 mM, *M*_3_ = 139 ± 12 mM without correction.

Figure 5 shows an example of sodium data quantification and filtering in an axial slice of the 3-compartment phantom, with and without frequency offset and ${B}_{1}^{+}$ correction. ROI measurements in the phantom compartments provide the following mean values ± standard deviations: *α*_1_ = 0.82 ± 0.12, *α*_2_ = 0.99 ± 0.11, *α*_3_ = 1.01 ± 0.07, *M*_1_ = 129 ± 30 mM, *M*_2_ = 139 ± 15 mM, *M*_3_ = 140 ± 10 mM when frequency offset and ${B}_{1}^{+}$ correction was included in the quantification, and *α*_1_ = 0.97 ± 0.13, *α*_2_ = 0.70 ± 0.12, *α*_3_ = 0.99 ± 0.09, *M*_1_ = 143 ± 29 mM, *M*_2_ = 97 ± 16 mM, *M*_3_ = 139 ± 12 mM without correction. Since the phantom was designed in a way that for each voxel, the signal from only one compartment was acquired, we would expect to measure volume fractions of ${\alpha }_{1}^{theory}=0.92\pm 0.02$, ${\alpha }_{2}^{theory}=0.96\pm 0.02$, and ${\alpha }_{3}^{theory}=1$, according to the three agar gel compartments (8%, 4% and 0% agar gel concentrations). The apparent sodium concentrations *M*_1_, *M*_2_, and *M*_3_ can be calculated according to equation 3 (see Methods): with ${\alpha }_{j}^{theory}$ as described above, we expect to measure ${M}_{1}^{theory}\mathrm{=128.8}\pm 7.4$ mM, ${M}_{2}^{theory}\mathrm{=134.4}\pm 7.6$ mM, and ${M}_{3}^{theory}\mathrm{=140}\pm 5$ mM, according to the known ^23^Na concentrations *C*_1_ = *C*_2_ = *C*_3_ = 140 ± 5 mM all over the phantom.

We can notice that frequency offset and ${B}_{{\bf{1}}}^{+}$ correction from simulation of a dictionary as described in “Methods\Data quantification processing” improved both the homogeneity and the results from the quantification in phantoms, mainly in 4% and 8% gels.

### Sodium quantification in healthy volunteers

**Figure 6:**
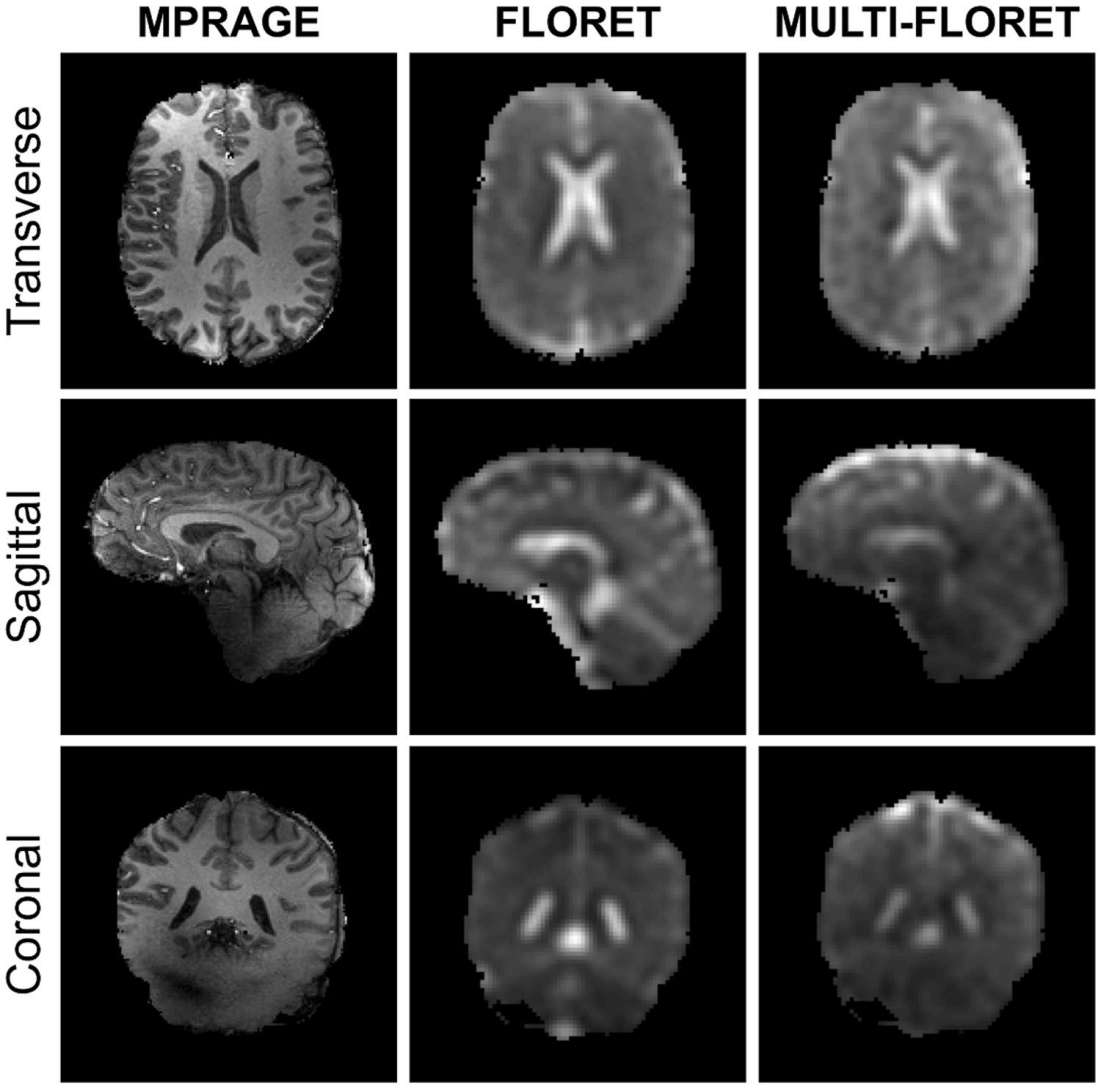
Examples of ^1^H and ^23^Na images in brain of a healthy volunteer: transverse, sagittal and coronal slices. The proton MPRAGE image was acquired with 1.25 mm isotropic resolution. The sodium (single) FLORET was acquired with 10 average and 5 mm isotropic resolution. The sodium multi-FLORET image is the sum of the 15 images acquired with the 15-pulse (multi) FLORET with 4 averages and 5 mm isotropic resolution. Both FLORET and multi-FLORET show comparable quality and resolution, but slightly different contrast due to the different contrast of each individual multi-FLORET acquisition. See Methods for acquisition details.

Figure 6 presents examples of brain images from one volunteer, acquired with ^1^H MPRAGE, ^23^Na single-FLORET and ^23^Na multi-FLORET, in transverse, sagittal and coronal planes. The multi-FLORET image presented here is the sum image of all 15 individual images of the 15-pulse multi-FLORET sequence, and is shown for comparison of image quality compared to single-FLORET. Both single-FLORET and multi-FLORET show comparable quality and resolution, but slightly different contrast due to the different contrast of each individual multi-FLORET acquisition.

**Figure 7:**
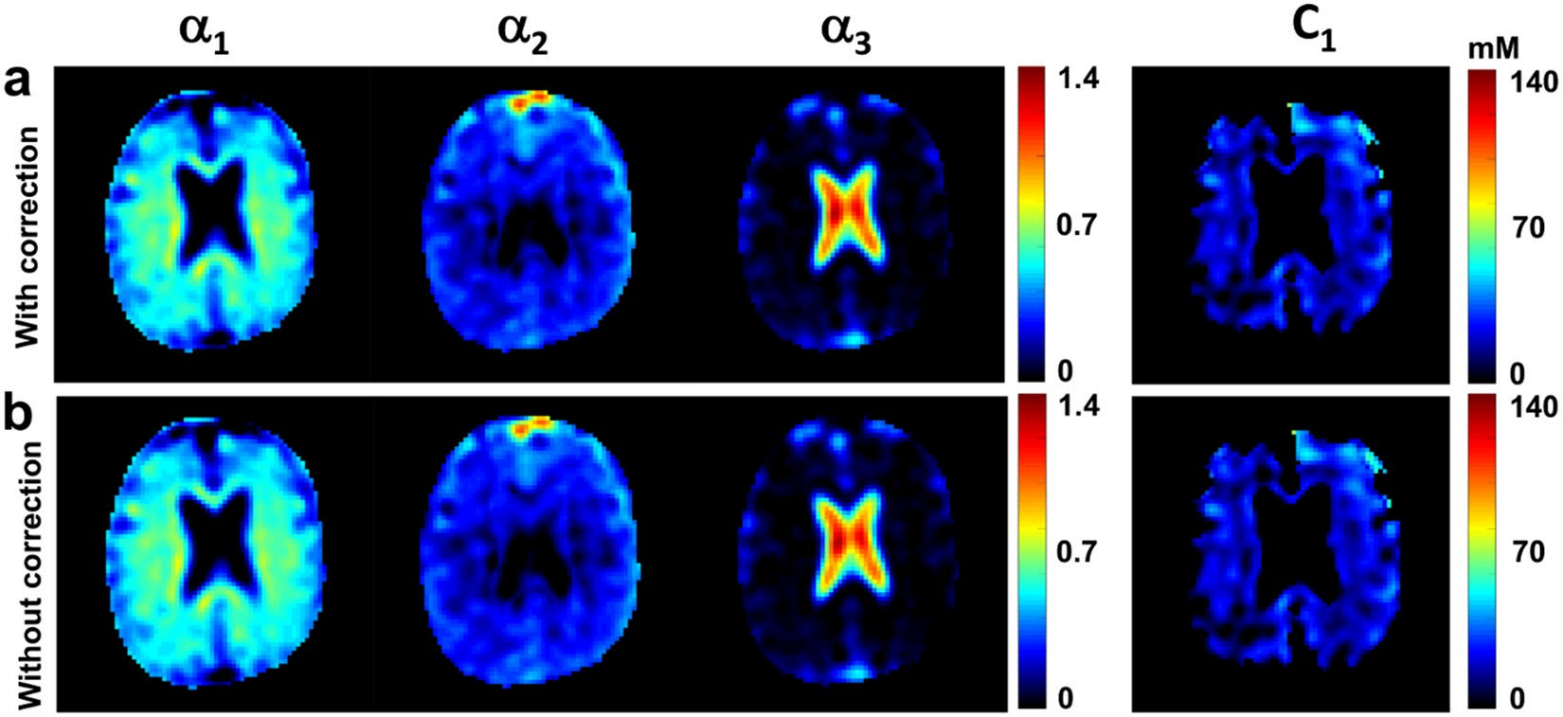
Example of sodium data quantification in brain in one volunteer. (**a**) The top row shows quantitative maps of *α*_1_, *α*_2_, *α*_3_, and *C*_1_ calculated with frequency offset and ${B}_{1}^{+}$ correction. (**b**) The bottom row shows quantitative maps of *α*_1_, *α*_2_, *α*_3_, and *C*_1_ calculated without correction. Evaluations of the same volume of interest for all quantified images yield mean values ± standard deviations of *α*_1_ = 0.55 ± 0.09, *α*_2_ = 0.22 ± 0.08, *α*_3_ = 1.01 ± 0.16, and *C*_1_ = 21 ± 8 mM with correction, and *α*_1_ = 0.57 ± 0.09, *α*_2_ = 0.20 ± 0.08, *α*_3_ = 1.01 ± 0.16, and *C*_1_ = 20 ± 8 mM without correction. Maps homogeneities and mean values measured with and without correction are not significantly different. See Methods for details of evaluations and calculations. See Table 3 for measurements in all volunteers.

Quantified maps of *C*_1_, *α*_1_, *α*_2_, and *α*_3_ from one volunteer are shown in Fig. 7, with and without frequency offset and ${B}_{1}^{+}$ correction (top and bottom rows respectively). The *α*_1_, *α*_2_, and *C*_1_ maps show good differentiation between CSF and parenchyma (gray and white matters). The *α*_3_ map primarily yields signal from the CSF brain compartment in ventricles and subarachnoid space. Evaluations in the same volume of interest in all quantified images generate mean values  ±  standard deviations of *α*_1_ = 0.55 ± 0.09, *α*_2_ = 0.22 ± 0.08, *α*_3_ = 1.01 ± 0.16, and *C*_1_ = 21 ± 8 mM with correction, and *α*_1_ = 0.57 ± 0.09, *α*_2_ = 0.21 ± 0.08, *α*_3_ = 1.02 ± 0.16, and *C*_1_ = 21 ± 8 mM without correction. Maps homogeneity and mean values measured with and without correction were not significantly different.

A list of measurements of *C*_1_, *α*_1_, *α*_2_, and *α*_3_ in the brain of four healthy volunteers is shown in Table 3. Mean values  ±  standard deviations of the mean values from all volunteers are: *α*_1_ = 0.54 ± 0.01, *α*_2_ = 0.23 ± 0.01, *α*_3_ = 1.03 ± 0.01, and *C*_1_ = 23 ± 3 mM.

**Table 3:** Quantification results in brain in four healthy volunteers for 15-pulse sequence. Results include volume fractions *α*_1_, *α*_2_, and *α*_3_, corresponding to intracellular, extracellular and CSF compartments, respectively, as well as intracellular ^23^Na concentrations *C*_1_ for four volunteers. Results are within the ranges for the expected physiological values of ${\alpha }_{1}^{theory}$ ∼ 0.5–0.6, ${\alpha }_{2}^{theory}$ ∼ 0.2–0.3, ${\alpha }_{3}^{theory}\sim 1$, and ${C}_{1}^{theory}$ ∼ 10–30 mM within standard deviations. All values in the table are given as mean ± standard deviations.

Volunteer	*α*_1_	*α*_2_	*α*_3_	*C*_1_ (mM)
1	0.55 ± 0.10	0.21 ± 0.09	1.06 ± 0.21	21 ± 8
2	0.52 ± 0.14	0.26 ± 0.16	1.05 ± 0.21	32 ± 14
3	0.55 ± 0.09	0.22 ± 0.08	1.01 ± 0.16	21 ± 8
4	0.55 ± 0.10	0.23 ± 0.10	1.00 ± 0.16	19 ± 8
Average	**0.54 ± 0.01**	**0.23 ± 0.01**	**1.03 ± 0.01**	**23 ± 3**

Uncertainty propagation from possible variations of sodium concentration in extracellular space and in CSF from the average value of 140 mM (used as reference in our method), with variations in the range  ± 10 mM, was simulated in ideal voxels of CSF or brain tissues, as described in the Methods/Uncertainty propagation section. Results are shown in Table 4. In summary, uncertainties of ±7% in *C*_2_ and *C*_3_ lead to uncertainties of ±13% in *α*_1_, ±5% in *α*_2_, ±7% in *α*_3_, and ±18% in *C*_1_.

**Table 4:** Uncertainty propagation for variations of sodium concentration in CSF and extracellular space. We simulated the quantification by assuming an ideal sample of two voxels with original values of *C*_1_, *α*_1_, *α*_2_ and *α*_3_: one voxel in CSF with ${\alpha }_{3}^{0}\mathrm{=1}$, and one voxel in brain tissue with ${C}_{1}^{0}\approx 15$ mM, ${\alpha }_{1}^{0}\mathrm{=0.6}$ and ${\alpha }_{2}^{0}\mathrm{=0.2}$, but with variable ${C}_{2}^{0}={C}_{3}^{0}\mathrm{=130}-150$ mM (as can occur *in vivo* due to pathologies or inter-subject variability). The quantification was performed as described in the “Multicompartment sodium quantification theory” section in Methods, assuming a constant value *C*_*e*_ = 140 mM for CSF and extracellular sodium concentration. Variation in the “real” values of *C*_2_ and *C*_3_ therefore lead to uncertainties in the calculation of *C*_1_, *α*_1_, *α*_2_ and *α*_3_.

	***C***_**2**_, ***C***_**3**_ (mM)	***C***_**1**_ (mM)	***α***_**1**_	***α***_**2**_	*α*_3_
	130	12.9	0.68	0.19	0.93
	135	14.0	0.64	0.19	0.96
Original values	**140**	**15.0**	**0.60**	**0.20**	**1.00**
	145	16.5	0.56	0.21	1.04
	150	18.2	0.52	0.21	1.07
Mean absolute uncertainty	±10	±2.7	±0.08	±0.01	±0.07
**Absolute uncertainty in %**	±**7%**	±**18%**	±**13%**	±**5%**	±**7%**

In Table 5, we compare the results in sodium quantification from different ^23^Na MRI methods developed by different groups with the results from this study, and with the expected theoretical values in healthy brain tissues taken from the literature. We can see that the proposed multipulse acquisition combined with a 4-compartment model of brain tissue generated for the first time values of all four parameters *C*_1_, *α*_1_, *α*_2_, and *α*_3_ within the range of the expected values in healthy tissue.

**Table 5:** Comparison of sodium quantification in brain from different studies. Values presented in this table are the average values in healthy brain tissue (white and/or gray matter) from the different references.

**Reference**	**TSC** (mM)	***C***_**1**_, **ISC, vBSC** (mM)	***α***_**1**_, **CVF, TCD, ISVF**	***α***_**2**_	*α*_3_	**Tissue model**	**Method**
Ouwerkerk *et al*.^13^	60–70^1^					1-CM	SQ
Boada *et al*.^55^	40–45					1-CM	SQ
Inglese *et al*.^34^	20–30					1-CM	SQ
Thulborn *et al*.^56^	28–38^2^, 40–46^3^		0.82–0.86			3-CM	SQ
Zaaraoui *et al*.^57^	40–63					1-CM	SQ
Fleysher *et al*.^58^		10–15	0.85–0.95			2-CM1	TQF
Paling *et al*.^59^	30–40					2-CM2	SQ
Qian *et al*.^60^	40–70	5–27				2-CM3	2SQ
Maarouf *et al*.^61^	37–52					1-CM	SQ
Mirkes *et al*.^62^	30–37					1-CM	SQ
Madelin *et al*.^18,63^		5–20		0.10–0.25		3-CM	IR
Niesporek *et al*.^64^	41–48					1-CM	SQ
Petracca *et al*.^65^	25–45	11–15	0.83–0.92			2-CM1	TQF
Thulborn *et al*.^66^	35–40		0.79–0.82			2-CM1	SQ
**This study**	**40–54** ^4^	**19–32**	**0.52–0.55**	**0.21–0.26**	**1.00–1.06**	**4-CM**	**MP**
**Theoretical values** ^1–10,23–28^	**33–48**	**10–30**	**0.50–0.60**	**0.20–0.30**	**0.99–1.00**		

Abbreviations: IC = intracellular compartment; EC = extracellular compartment; CSF = cerebrospinal fluid; TSC = total sodium concentration; *C*_1_^18,63^ (present study) = ISC^58^ = intracellular sodium concentration; vBSC^60^ = volume-fraction weighted bound sodium concentration; *α*_1_^18,63^ (present study) = intracellular volume fraction; CVF^66^ = cell volume fraction; TCD^56^ = tissue cell density; ISVF^58^ = intracellular sodium volume fraction; *α*_2_^18,63^ (present study) = extracellular volume fraction; *α*_3_ (present study) = CSF volume fraction; 1-CM = 1-compartment model (average IC + EC); 2-CM1 = 2-compartment model (IC, EC); 2-CM2 = 2-compartment model (tissue, CSF); 2-CM3 = 2-compartment model (bound sodium = IC, fluid sodium); 3-CM = 3-compartment model (IC, EC, water fraction/solid space); 4-CM = 4-compartment model (IC, EC, CSF, water fraction/solid space); SQ = single quantum; 2SQ = 2 single quantum; TQF = triple quantum filter, MP = multi-pulse; IR = inversion recovery.

Notes: ^1^Assuming brain density = 1 for conversion from mmol/kg to mM. ^2^Not including water fraction. ^3^Including water fraction. ^4^TSC = *α*_1_ *C*_1_ + *α*_2_ *C*_2_, with *C*_2_ = 140 mM.

## Discussion

The quantified magnetizations *M*_1_, *M*_2_, and *M*_3_ as a measure of the apparent sodium concentrations show a good distinction between the three phantom compartments, demonstrating the method’s applicability as a filtering technique for compartments with different relaxation times. The measured *M* values in the three phantom compartments were close to the theoretical values when frequency offset and ${B}_{1}^{+}$ correction were included in the quantification process, but with limited accuracy due the standard deviations in the range 10–30 mM. These high standard deviations in the quantification *M*_*i*_ (i = 1 to 3) are mostly due to the low SNR in some images (i = 6, 13,14, 15) and to the non-optimal shimming on the 3-compartment phantom (probably caused by many interfaces between the gel containers used for each compartment, both in transverse orientation, but also along the magnetic field where the bottom and top of the containers may contain some air). The decorrelation of signal from 4% and 8% agar gel compartments proved difficult because these two compartments have relaxation times that are closer to each other, and the correlation could not be reduced below to 0.7 in our current optimization process. This relatively high correlation between the two signals from 4% and 8% agar gels also adds uncertainty in the quantification of the *M*_*i*_ values. The signal from the 0% agar gel compartment, on the other hand, was easy to decorrelate from the signals from the other compartments, as its T_1_ is considerably longer and its ${{\rm{T}}}_{2}^{\ast }$ relaxation is monoexponential and close to T_1_.

However, the fundamental purpose of this study was to decorrelate the signal evolutions from different brain *in vivo* compartments and to test the applicability of the model in phantom measurements. Therefore, the present 15-pulse sequence was optimized for brain compartments. Sodium ions in the 8%, 4%, and 0% agar gel compartments are considered to show sodium relaxation times that are roughly similar to the relaxation times in the IC, EC, and CSF compartments in brain, respectively. The relaxation time differences between the IC and EC compartment, however, are larger than those between the 4% and 8% agar gel compartments. The signal deriving from IC and EC compartment can therefore be decorrelated more efficiently with the present 15-pulse sequence. For reasons of consistency, the same 15-pulse sequence was applied for phantom and brain experiments. Despite some inaccuracies, the phantom quantification still yields sharply separated compartments and the mean values of volume fractions *α* and *M* correspond with theoretically expected values within standard deviations.

To evaluate the reliability of the simulated data, and thus the *λ* matrix, for the phantom quantifications, we looked at the correlation coefficients between the simulated and the measured phantom signal developments. The 4% and 8% gel compartments show a high correlation coefficient of 0.95 between simulated and measured signal evolutions, while it is even higher (0.99) for the 0% gel (fluid). These high correlation results validate the accuracy of the program for simulating sodium the spin 3/2 dynamics during a multipulse RF sequence.

Brain quantification results also show the method’s ability to separate different compartments *in vivo*. In theory, distributions of ${\alpha }_{1}^{theory}$ ∼ 0.5–0.6^23,26–28^, ${\alpha }_{2}^{theory}$ ∼ 0.2–0.3^25^, and intracellular ^23^Na concentration ${C}_{1}^{theory}$ ∼ 10–30 mM^1–10^ in gray matter (GM) and white matter (WM) were expected in healthy brain. We also expect the the CSF volume fraction to be ${\alpha }_{3}^{theory}=0$ in GM and WM (except in voxels close to CSF compartments due to partial volume effects), and ${\alpha }_{3}^{theory}\sim 1$ in the CSF compartments (ventricles and subarachnoid spaces). Overall, results for *α*_1_, *α*_2_, *α*_3_, and *C*_1_ in brain *in vivo* correspond with expected physiological values within standard deviations. Inaccuracies in the *C*_1_ maps mainly occur in the transition areas between the white and gray matters and the lateral ventricles, and are likely due to partial volume effects. Applying a mask based on the *α*_3_ map can remove these inaccuracies (as shown in Fig. 7). Another source of inaccuracy is the spatial variations in the water fraction between GM and WM (*w*_*GM*_ ∼ 0.85, *w*_*WM*_ ∼ 0.7)^45^. In this pilot study, a water fraction of *w* = 0.8 was assumed for the whole brain tissue as a first approximation. This assumption will be corrected in our future work by including a water fraction measurement from proton MRI^29,30^, which was not included at present due to difficulties in water fraction quantification at 7 T related to B_0_ and B_1_ inhomogeneities in ^1^H MRI.

Uncertainties in *C*_1_, *α*_1_, *α*_2_ and *α*_3_ quantification can also occur due to variations of *C*_2_ and *C*_3_ away from the value *C*_*e*_ = 140 mM, which was assumed constant in CSF and extracellular space in our model. These changes in *C*_2_ and *C*_3_ can be due to inter-subject differences, to chronobiology^46^, and pathologies such as migraine^47^, with changes generally in the range 130–150 mM. We see from simulation that these variations of *C*_2_ and *C*_3_ (of about ±7%) lead to uncertainties in *C*_1_, *α*_1_, *α*_2_ and *α*_3_ that are smaller than the standard deviations of the measurement in each individual subject (see Tables 3 and 4). These uncertainties should however be taken into account when performing quantification with the proposed method as it is, and changes measured in *C*_1_, *α*_1_, *α*_2_ and *α*_3_ below the uncertainty values should be considered as non-significant. In a future study on repeatability and reproducibility of the method, we are planning to compare the use of a constant CSF sodium concentration as a reference with the use of an external phantom of known sodium concentration and relaxation times, and known water fraction, placed next to the brain during the MRI scan.

The multicompartment ^23^Na quantification, as the result of multiple signal acquisitions, can be sensitive to *B*_0_ inhomogeneities, which lead to phase shifts and quantification inaccuracies. As the multicompartment ^23^Na quantification signal also depends on flip angles *θ*_*i*_, ${B}_{1}^{+}$ (transmit) inhomogeneities can further deteriorate the image quality of the quantified ^23^Na images. Receive ${B}_{1}^{-}$ inhomogeneities can also influence the quantification. However, applying a uniform phantom correction before data quantification did not improve results in our case, as the birdcage RF coil used in our study was already producing a uniform signal reception over the samples. The phantom images of the 15-pulse sequence indicate signal inhomogeneities for acquisitions after relatively large flip angle pulses (for example after the second RF pulse with flip angle *θ*_2_ = 150°). Limitations of flip angles to smaller values thus seem desirable for signal optimization with good spatial homogeneity, and to limit SAR. At the same time, a sufficiently large flip angle variability seems necessary for the decorrelation of signals deriving from the different compartments. The present 15-pulse sequence can be seen as a trade-off between these two requirements. It gave reasonable results in phantom experiments (where filtering and quantification tests are possible) despite some spatial inhomogeneities, and it could generate a sufficient decorrelation of the signals from different brain compartments to allow a reasonable sodium quantification *in vivo*. In brain experiments, inhomogeneities were actually less severe due to better shimming and lower reference voltages.

In our approach, we did not measure *B*_0_ and ${B}_{1}^{+}$ maps, as correcting the data with these maps would necessitate to recalculate the *λ* matrix for each voxel of the 3D data, which involves a full simulation of all ISTOs from all compartments while including local frequency offsets and ${B}_{1}^{+}$ inhomogeneities measured with the maps. This correction method was deemed too time consuming for our limited computing power. We decided instead to simulate a dictionary of signal evolutions in CSF or 0% agar gel over the multipulse sequence with different frequency offsets and ${B}_{1}^{+}$ inhomogeneities (see Methods\Data quantification processing), and compare the signal evolutions from this dictionary with the signal evolution that can be measured in the 0% gel and in CSF in the ventricles (where it is assumed to be a single compartment where only CSF is present). The entry from the dictionary that gives the highest correlation with the measured signal evolution therefore provides us with an average frequency offset and ${B}_{1}^{+}$ correction factor that can then be applied to the *λ* matrix entries for IC and EC (or 4% and 8% agar gels). This self-correction method was successfully tested on the phantom data and in brain *in vivo*, as seen in Figs 5 and 7. The effect of this correction was more evident on the phantom quantification, where the shim was sub-optimal and the correlation between the signals of the 4% and 8% gels was still high after optimization (0.7), leading to values of volume fractions and apparent sodium concentrations closer to the expected theoretical values when corrected. Results in brain were less affected by the correction, thanks to a better shim in brain and better decorrelation between IC and EC signal evolutions. The mean values of *C*_1_, *α*_1_, *α*_2_ and *α*_3_ measured with and without correction were all in the range of the expected theoretical values.

The proposed quantification method is based on a multicompartment model of brain tissue, where we assume that sodium ions are only present in the IC, EC and CSF compartments of the human brain, and that these compartments can be distinguished by their different ^23^Na relaxation times. According to this model, the total sodium signal for a brain MRI corresponds to the weighted sum of the signals from these three tissue compartments, characterized by the concentrations *C*_*j*_, volume fractions *α*_*j*_, and weighting factors *λ*_*j*_ in the corresponding volumes, with *j* = 1 (IC), 2 (EC) and 3 (CSF). The acquisition of N signals with a N-pulse FLORET sequence allows us to calculate *C*_1_, *α*_1_, *α*_2_, and *α*_3_. In this study, we use relaxation times from the literature for the brain signal simulations. Relaxation times for IC and EC spaces were taken from a brain tumor study in rats using shift reagents^15^. The study provides T_1_, ${{\rm{T}}}_{2l}^{\ast }$ and ${{\rm{T}}}_{2s}^{\ast }$ relaxation times assuming a monoexponential T_1_ and a biexponential ${{\rm{T}}}_{2}^{\ast }$ relaxation, according to our model in this study. Relaxation times for physiological human tissue can differ from those in tumor tissue in rats. However, Winter *et al*.^15^ investigate the sodium concentrations in their study, yielding *C*_2_ = 149 mM and *C*_1_ = 19 mM. The sodium environment appears to be similar to physiological human brain tissue and thus to the assumptions in the present multicompartment model. We compared the relaxation times by Winter *et al*.^15^ for the intracellular compartment with a collection of other ^23^Na intracellular relaxation times out of four different studies^41–43^ (see Table 6). Mean values of all sets of relaxation times T_1_, ${{\rm{T}}}_{2l}^{\ast }$ and ${{\rm{T}}}_{2s}^{\ast }$ are in good agreement with the relaxation times from Winter *et al*.^15^. We couldn’t compare extracellular ^23^Na relaxation times to other studies. One future goal of this project is to do further research into *in vivo* ^23^Na relaxation times. CSF relaxation times were taken from relaxation time measurements in human healthy volunteers by Nagel *et al*.^48^.

**Table 6:** Intracellular ^23^Na relaxation times from the literature. Abbreviations for T_2_: ${{\rm{T}}}_{2{\rm{l}}}^{\ast }$ = long ${{\rm{T}}}_{2}^{\ast }$, ${{\rm{T}}}_{2s}^{\ast }$ = short ${{\rm{T}}}_{2}^{\ast }$.

Reference	T_1_ (ms)	${{\rm{T}}}_{2l}^{\ast }$ (ms)	${{\rm{T}}}_{2s}^{\ast }$ (ms)
Shinar *et al*. 1991 (human erythrocytes)^41^	21.1 ± 0.1	14.8 ± 0.3	5.1 ± 0.4
Bansal *et al*. 1993 (rat liver)^42^	21.1 ± 0.6	13.0 ± 0.9	1.3 ± 0.1
Foy *et al*. 1990 (frog heart)^43^	22.4 ± 3.0	16.4 ± 4.2	2.0 ± 1.3
Foy *et al*. 1990 (rat heart)^43^	23.0 ± 2.5	19.0 ± 1.1	2.6 ± 0.8
Winter *et al*. 2001 (rat brain tumor)^15^	24.0 ± 4.0	13.9 ± 0.9	2.0 ± 0.8
Pettegrew *et al*. 1984 (human erythrocytes)^44^	30.0 ± 3.0		
**Average**	23.6 ± 1.4	15.4 ± 1.1	2.6 ± 0.7

Quantification results in brains of healthy volunteers correspond with the expected physiological values for IC and EC compartments. Therefore, the results do not rule out the physiological IC and EC compartments as source of the signal, despite the limitations and errors of the quantification method related to inaccuracies of the relaxation times.

The low SNR for the 15 individual sodium images can limit the accuracy of the data quantification, mainly *in vivo* where IC sodium concentrations are around 10–30 mM and can be difficult to evaluate with precision. However, the quantification can be more robust to noise by using more RF pulses (but at the expense of longer total acquisition times and higher SAR), by increasing the number of data averages, or using lower resolutions and higher magnetic fields.

Sequence optimizations allow flip angle and phase variations over the N-pulse sequence. Delay times between the RF pulses were set constant to *τ*_*i*_ = 5 ms in our pilot study, as variable delays did not provide better decorrelation between IC and EC. Flip angles *θ*_*i*_ were ranging from 0° to 150°. The restriction “*θ*_*i*_ ≤ 150°” was chosen to avoid high RF inhomogeneities due to high flip angles, and to keep the specific absorption rate (SAR), which increases with the number of pulses and may induce the necessity of using a long TR, within reasonable limits for *in vivo* acquisitions (<1 hour). The total scan time could subsequently be reduced to $\sim 9$ min per average.

As we can see in Table 5 where we compared different sodium MRI methods to quantify the sodium content and volume fractions in brain *in vivo*, most of the previous studies were focusing on total sodium concentration (TSC). TSC measurement cannot differentiate the influence of the intracellular sodium concentration from the volume fractions (intracellular or extracellular) when changes of sodium content are observed in MRI due to pathologies or treatments. Moreover, other studies aiming at distinguishing the two metrics *C*_1_ and *α*_1_ (or *α*_2_) were based on more simple tissue models (1 to 3 compartments) which often do not take into account the solid compartment where no sodium is present (or in negligible amount) and were based on only the T_1_ or the (biexponential) T_2_ difference between compartments. These studies also assumed that the sodium fluid in the extracellular compartment was similar to the CSF, with monoexponential T_2_ ∼ T_1_. As clearly explained in Thulborn 2016^22^, the extracellular space can also include bound sodium similarly to the intracellular space, due to the presence of macromolecules and restricted volume fraction, and therefore extracellular sodium possess biexponential T_2_, with T_2s_ and T_2l_ < T_1_. The EC must then be considered as a separate fluid compartment that can generate TQF signal, for example, or residual signal when applying fluid suppression by inversion recovery that was optimized for CSF. In our multipulse method associated with a 4-compartment model, we tried to extract the contribution from three brain tissue compartments of interest (IC, EC, CSF) using both sodium T_1_ and T_2_ relaxation parameters from these compartments.

The approach to use simulated signal evolutions for signal analyses is inspired by magnetic resonance fingerprinting (MRF)^49^. However, since ^23^Na is a spin 3/2 nucleus with four quantized energy states, Bloch equations cannot be used for simulations of quadrupolar spin dynamics. Instead, the simulated signal for spin 3/2 ^23^Na nuclei was calculated by including the developments of the complete set of 16 irreducible spherical tensor operators (ISTO) (see Methods for details), in order to take into account the presence of double and triple quantum coherences that can be created and can evolve during the different RF pulses and delays, and that can influence the detected signal after each pulse. Besides computational differences in the simulation procedure, there are also methodological differences compared to MRF. In our case, we assumed that we knew the approximate sodium relaxation times in different compartments in the brain (IC, EC, CSF). We then tried to deduce their respective contribution to the final signal using their simulated signals over a N-pulse sequence (N = 15 in this study) as a basis for the total signal decomposition. The metabolic parameters of interests (*C*_1_, *α*_1_, *α*_2_, and *α*_3_) were then calculated by simple matrix inversion (see Methods). Applying the usual MRF method in brain to measure T_1_, T_2*l*_, and T_2*s*_ (and their corresponding weighting coefficients C_T__2*l*_ and C_T__2*s*_) proves to be more difficult, as there are five parameters to fit, with a limited range (relaxation times of sodium are very short compared to those of proton, with T _1_ and T _2_ < 100 ms), and low SNR of sodium images.

The proposed method could prove useful as a new non-invasive imaging technique to investigate *in vivo* loss of ion homeostasis in pathologies related to changes in sodium ions concentrations in the intracellular and extracellular spaces, and changes in their respective volume fractions. Clinical applications such as evaluation of tumor malignancy, cancer therapy monitoring, or longitudinal assessment of neurodegeneration in early Alzheimer’s disease, in multiple sclerosis or traumatic brain injury might benefit from such a quantitative imaging method. A future objective will be to substantially reduce acquisition time through more efficient sequences that may allow a lower number of RF pulses with lower flip angles *θ*_*i*_, combined with undersampling acquisitions and compressed sensing reconstruction^50,51^.

In conclusion, in this preliminary study, we showed that a multipulse sequence for ^23^Na data acquisition along with simulation of ^23^Na signal evolution over this sequence at 7 T can differentiate different compartments in phantom and in healthy human brain *in vivo* and provide an estimate of intracellular sodium concentration as well as intracellular, extracellular and CSF volume fractions.

## Methods

### Human subjects

The method was tested *in vivo* for brain scans in four healthy volunteers (2 males, 2 females, mean age = 26 ± 2 years) after approval from the Institutional Review Board of New York University School of Medicine and signed inform consent, and in accordance with Food and Drugs Administration guidelines.

### MRI hardware

MRI experiments were performed on a 7 T whole-body MR system (Magnetom 7 T, Siemens, Erlangen, Germany) using a custom-built dual-tuned transmit/receive ^1^H/^23^Na radiofrequency birdcage head coil with 27.9 cm inner diameter.

### Phantom fabrication

In order to simulate different tissue sodium compartments in phantom measurements, sodium chloride (NaCl) was embedded in gels with variable agar gel concentrations. A cylindrical phantom (3200-ml cylinder, 16.8 cm diameter, 30.0 cm height) consisting of three different concentric compartments was built, as shown in Fig. 1a. All compartments were filled with NaCl solution (140 ± 5 mM) containing different agar gel concentrations (in cross section: 0% agar in the center, 8% agar in the inner ring, 4% agar in the outer ring). Gel concentrations of 0%, 4% and 8% were chosen such as they can approximately model the relaxation times of ^23^Na ions in the CSF, the extracellular and the intracellular compartments in the brain, respectively, which are considered to have similarly restricted mobility. Theoretical expected values for volume fractions and ^23^Na concentrations for all three phantom compartments are: *C*_1_ = *C*_2_ = *C*_3_ = 140 ± 5 mM, ${\alpha }_{1}^{theory}\mathrm{=0.92}\pm 0.02$ (8% gel), ${\alpha }_{2}^{theory}\mathrm{=0.96}\pm 0.02$ (4% gel), and ${\alpha }_{3}^{theory}\mathrm{=1}$ (0% gel). Uncertainties were estimated for inaccuracies in the phantom fabrication (weight measurement of agar powder, water volume used).

Note that the agar gel phantoms (0%, 4% and 8%) reflect only roughly the relaxation times of the CSF, EC and IC compartments, but not the sodium concentrations. The goal of using this 3-compartment phantom was mainly to test the validity of the method for differentiating multiple compartments based on their different relaxation times only, without being influenced by differences in contrast due to different sodium concentrations. We tried to make gels with relaxation times similar to the relaxation times in CSF, EC and IC to have an estimate of the signal differentiation between compartments in brain that we can expect with this method. A concentration of 140 mM in the gels was chosen to generate sufficient SNR and a higher resolution than *in vivo* data (3.6 mm for phantom vs. 5 mm for brain).

### Multicompartment tissue model

A four-compartment model of brain tissue (Fig. 1b) was proposed, where sodium ions are only present in the IC, EC, and CSF compartments of the human brain, labeled as compartments 1, 2, and 3, respectively. Compartment 4 represents all solid tissue components (membranes, lipids, and other molecules and organelles) not containing any sodium, and therefore it does not contribute to the sodium signal detected by MR but it still occupies some space in tissue that needs to be included in the calculations of different volume fractions in our model. In this preliminary model, the vascular space, which occupies around 3% of brain volume^31^, was assumed to be negligible and part of the extracellular compartment. The EC compartment therefore includes the interstitial and vascular spaces. The model was used to develop the quantification of intracellular ^23^Na concentration (*C*_1_, in mmol/L, or mM), as well as IC, EC, and CSF volume fractions *in vivo* (*α*_1_, *α*_2_, and *α*_3_, respectively). Sodium ions are present in three compartments in brain with concentrations *C*_1_, *C*_2_ and *C*_3_. The compartments *j* (with *j* = 1, 2 and 3) described by volumes *V*_*j*_ are considered as fractions of the non-solid part of the tissue with volume fractions *α*_*j*_ = *V*_*j*_/*V*_*total*_ (*V*_*total*_ is the total voxel volume including both fluid and solid components).

The non-solid part of the tissue as a whole is characterized by the water fraction *w*, that corresponds in our case to the sum of the volume fractions, *w* = *α*_1_ + *α*_2_ + *α*_3_. The extracellular ^23^Na concentrations *C*_2_ and *C*_3_ in the model are set to be constant at 140 mM^1,2^. The water fraction in our brain tissue model was assumed to be constant *w* = 0.8 in this pilot study, which corresponds to an averaging of expected *w* values in GM and WM^45^. The unknown variables in the four-compartment model are *C*_1_, *α*_1_, *α*_2_, and *α*_3_.

### Multicompartment sodium quantification theory

The total ^23^Na quantified total signal *S* in this model corresponds to the sum of the ^23^Na signal from three tissue compartments, depending on the concentrations *C*_*j*_, volume fractions *α*_*j*_, and weighting factors *λ*_*j*_ (with *j* = 1, 2 and 3), for the different compartments after one RF pulse:

1 $$S=(\begin{array}{ccc}{\lambda }_{1} & {\lambda }_{2} & {\lambda }_{3}\end{array})(\begin{array}{c}{C}_{1}{\alpha }_{1}\\ {C}_{2}{\alpha }_{2}\\ {C}_{3}{\alpha }_{3}\end{array})\mathrm{.}$$

To be able to solve this system of equations for the unknown values *C*_1_, *α*_1_, *α*_2_, and *α*_3_, the system is extended as follows,

2 $$(\begin{array}{c}{S}_{1}\\ {S}_{2}\\ \vdots \\ {S}_{N}\end{array})=(\begin{array}{ccc}{\lambda }_{11} & {\lambda }_{12} & {\lambda }_{13}\\ {\lambda }_{21} & {\lambda }_{22} & {\lambda }_{23}\\ \vdots  & \vdots  & \vdots \\ {\lambda }_{N1} & {\lambda }_{N2} & {\lambda }_{N3}\end{array})\,(\begin{array}{c}{C}_{1}{\alpha }_{1}\\ {C}_{2}{\alpha }_{2}\\ {C}_{3}{\alpha }_{3}\end{array})$$

with *S*_*i*_ = signal acquisitions from multiple pulses *i* = 1 to N, and ${\lambda }_{ij}\equiv {\lambda }_{ij}({\theta }_{i},{\phi }_{i},{\tau }_{i},{T}_{1},{T}_{2l}^{\ast },{T}_{2s}^{\ast },TE,TR)=$ simulated maximum absolute signal (center of k-space) after pulse *i* for unit magnetization and unit volume fraction with relaxation from multiple compartments *j* = 1, 2 and 3.

Introducing a term for the vector “magnetization” *M* (where each element of *M* corresponds to the apparent sodium concentration in each compartment),

3 $$M=(\begin{array}{c}{M}_{1}\\ {M}_{2}\\ {M}_{3}\end{array})=(\begin{array}{c}{C}_{1}{\alpha }_{1}\\ {C}_{2}{\alpha }_{2}\\ {C}_{3}{\alpha }_{3}\end{array}),$$

the quantified total sodium signal *S* can be described as the product of the *λ* matrix with the magnetization *M*,

4 $$S=\lambda M\mathrm{.}$$

Assuming that we can measure the signal *S* and simulate the *λ* matrix (see below, in “Multicompartment signal simulation” section), equation 4 can be solved for *M*, and therefore for the unknown values *C*_1_, *α*_1_, *α*_2_, and *α*_3_:

5 $$M={\lambda }^{-1}S={({\lambda }^{H}\lambda )}^{-1}{\lambda }^{H}S$$

6 $${\alpha }_{2}=\frac{{M}_{2}}{{C}_{e}}$$

7 $${\alpha }_{3}=\frac{{M}_{3}}{{C}_{e}}$$

8 $${\alpha }_{1}=w-\frac{{M}_{2}+{M}_{3}}{{C}_{e}}$$

9 $${C}_{1}=\frac{{M}_{1}}{{\alpha }_{1}}=\frac{{M}_{1}{C}_{e}}{w{C}_{e}-{M}_{2}-{M}_{3}},$$

with *C*_2_ = *C*_3_ = *C*_*e*_ = 140 mM for the constant EC and CSF extracellular sodium concentration, and *λ* ^*H*^ = the Hermitian of *λ*.

### Uncertainty propagation

In our model, we assumed that the sodium concentrations in the extracellular compartment and in CSF were constant and equal to an average value *C*_2_ = *C*_3_ = *C*_*e*_ = 140 mM. However, this concentration can slightly change between individuals, the time of the day^46^, and pathologies such as migraine^47^, with changes generally in the range 130–150 mM. To estimate the uncertainty of the sodium quantification due to potential variations of *C*_2_ and *C*_3_ away from the average 140 mM value, we re-calculated the values of *C*_1_, *α*_1_, *α*_2_ and *α*_3_ in one ideal voxel in CSF with “true” value ${\alpha }_{3}^{0}=1$, and in one ideal voxel in brain tissue with “true” values ${C}_{1}^{0}=15$ mM, ${\alpha }_{1}^{0}=0.6$ and ${\alpha }_{2}^{0}=0.2$, but all with variable ${C}_{2}^{0}={C}_{3}^{0}=130-150$ mM. The quantification was performed as described in the “Multicompartment sodium quantification theory” section in Methods, keeping a constant value *C*_*e*_ = 140 mM for CSF and extracellular sodium concentration in the calculations. Variation in the real *in vivo* values of *C*_2_ and *C*_3_ therefore lead to uncertainties in the calculation of *C*_1_, *α*_1_, *α*_2_ and *α*_3_, which are presented in Table 4.

### Multicompartment signal simulation

Sodium nuclei ^23^Na possess a total spin *I* = 3/2. Placed into a static homogeneous magnetic field B_0_ in z direction, these spins can be characterized by four quantized energy states, $|-\frac{3}{2}\rangle ,|-\frac{1}{2}\rangle ,|+\frac{1}{2}\rangle ,|+\frac{3}{2}\rangle \mathrm{.}$ The standard Bloch equations formalism for spin-1/2 systems cannot describe completely the spin-3/2 dynamics. Instead, using the irreducible spherical tensor operators (ISTO) *T*_*lm*_ formalism becomes necessary (see Table 7). In the case of spin 3/2, we need a set of 16 ISTOs, each of them contributing to the signal evolution. The density operator *ρ* representing the spin ensemble can be decomposed in ISTOs as follows,

10 $$\rho =\sum _{ij}{\rho }_{ij}|i\rangle \langle j|=(\begin{array}{cccc}{\rho }_{11} & {\rho }_{12} & {\rho }_{13} & {\rho }_{14}\\ {\rho }_{21} & {\rho }_{22} & {\rho }_{23} & {\rho }_{24}\\ {\rho }_{31} & {\rho }_{32} & {\rho }_{33} & {\rho }_{34}\\ {\rho }_{41} & {\rho }_{42} & {\rho }_{43} & {\rho }_{44}\end{array}),$$

11 $$\rho =\sum _{l,m}{c}_{lm}{T}_{lm},$$

with the spin states $|i\rangle ,|j\rangle \in \{|1\rangle \equiv |+\frac{3}{2}\rangle ,|2\rangle \equiv |+\frac{1}{2}\rangle ,|3\rangle \equiv |-\frac{1}{2}\rangle ,|4\rangle \equiv |-\frac{3}{2}\rangle \}$, and weighting factors (complex numbers) *c*_*lm*_, with *l* = 0, 1, 2 or 3, and *m* = *−l, −l* + *1,…, l−1, l*.

**Table 7:** Irreducible spherical tensor operators (ISTOs). This table shows the relationship between ISTOs *T*_*lm*_ (*l* = 0 to 3 by steps of 1, and *m* = −*l* to *l* by steps of 1), and Cartesian spin operators. The set of 16 ISTOs is used for spin-3/2 sodium signal simulations in multicompartment ^23^Na quantifications. Acquired MR signal are represented by the *T*_1±1_ operator. *Abbreviations:* SQC, DQC and TQC stand for single, double and triple quantum coherences, respectively. The anticommutator for the operators A and B is defined as [A,B] _+_  = A × B + B × A.

*T*_*lm*_	Cartesian Decomposition	Description
*T*_00_	1	Identity
*T*_10_	*I*_*z*_	Longitudinal Magnetization
*T*_1±1_	$\mp \frac{1}{\sqrt{2}}{I}_{\pm }$	Rank 1 SQC
*T*_20_	$\frac{1}{\sqrt{6}}\mathrm{(3}{I}_{z}^{2}-I(I+\mathrm{1))}$	Quadrupolar Order
*T*_2±1_	$\mp \frac{1}{\sqrt{2}}{[{I}_{z},{I}_{\pm }]}_{+}$	Rank 2 SQC
*T*_2±2_	$\frac{1}{2}{I}_{\pm }^{2}$	Rank 2 DQC
*T*_30_	$\frac{1}{\sqrt{10}}\mathrm{(5}{I}_{z}^{3}-\mathrm{(3}I(I+\mathrm{1)}-\mathrm{1)}{I}_{z})$	Octupolar Order
*T*_3±1_	$\mp \frac{1}{4}\sqrt{\frac{3}{10}}{\mathrm{[5}{I}_{z}^{3}-I(I+\mathrm{1)}-\frac{1}{2},{I}_{\pm }]}_{+}$	Rank 3 SQC
*T*_3±2_	$\frac{1}{2}\sqrt{\frac{3}{4}}{[{I}_{z},{I}_{\pm }^{2}]}_{+}$	Rank 3 DQC
*T*_3±3_	$\mp \frac{1}{2\sqrt{2}}{I}_{\pm }^{3}$	Rank 3 TQC

The time evolution of the density operator *ρ* is then described by the Liouville-von Neumann equation (or master equation):

12 $$\frac{d}{dt}\rho (t)=-i[H,\rho (t)]-\hat{{\rm{\Gamma }}}(\rho (t)-{\rho }^{th}),$$

with total Hamiltonian *H* (which is the sum of all Hamiltonians acting on the density operators, such as Zeeman *H*_*Z*_, quadrupolar coupling *H*_*Q*_, and radiofrequency *H*_1_), relaxation superoperator $\hat{\Gamma }$ (based on the Redfield relaxation formalism^52,53^), and the density operator in thermal equilibrium *ρ* ^*th*^. Details about the Hamiltonians, relaxation superoperator and density operator evolution are described in the review by Madelin *et al*.^54^. The relaxation of spin 3/2 nuclei is dominated by the quadrupolar interaction between the nuclear electric quadrupole moment and the fluctuating local electrostatic field gradients. The measured signal in NMR corresponds to the single quantum coherences. The part of the signal to be simulated is thus represented by the average of the *T*_1±1_ operator (transverse magnetization), which is equal to the trace of the product of the density operator *ρ* with *T*_1±1_:

13 $$\langle {T}_{1\pm 1}\rangle =Tr(\rho {T}_{1\pm 1}\mathrm{).}$$

Based on these theoretical principles, sodium signal evolutions were simulated for the three compartments of interest (IC, EC, CSF) in our a four-compartment model (the solid space has no sodium signal). The simulated signal evolutions that were used in our quantification method were the maximum magnitude signals after each pulse. They correspond to the center of the k-space of each image and thereby represent the total signal of the sample. Parameters considered in the simulations included both sequence-specific parameters (*θ*_*i*_, *ϕ*_*i*_ and *τ*_*i*_, TE, TR), as well as compartment-specific parameters (T_1_, ${{\rm{T}}}_{2l}^{\ast }$, ${{\rm{T}}}_{2s}^{\ast }$). The simulation assumed no exchange between compartments. Different simulation dwell times were tested (20, 50, 100, 200 *μ*s), with no noticeable differences in the simulation results. The simulation dwell time was therefore set to 200 *μ*s to allow fast calculation (within a few tens of seconds), as simulations have to be re-performed after data acquisition to generate a dictionary of signal evolutions including a range of offset frequencies and RF transmit inhomogeneities (see the subsection Quantification Data Processing/Correction steps below).

Figure 3 shows an example of the evolution of ISTOs *T*_*lm*_ for the ^23^Na signal from the IC compartment during a 15-pulse sequence. The set of 15 tensor operators (*T*_00_ Identity is not shown) and their developments over the sequence are the basis for the simulated ^23^Na signal, represented by the maximum of the *T*_1±1_ operator, detected after each RF pulse. In order to imitate the measured data acquisition with TE = 0.4 ms (which would correspond to the center of k-space of the images acquired with a center-out non-Cartesian trajectory such as FLORET, and therefore correspond to their maximum signal), the maximum simulated signal after each RF pulse was detected after a 400 *μ*s delay, equivalent to the TE used for data acquisitions. Examples of simulated maximum absolute signal (center of k-space) are shown in Fig. 4 for a 15-pulse sequence.

All simulations were performed in Matlab (MathWorks, Natick, MA, USA).

### Phantom relaxation times

Sodium relaxation times for the three phantom compartments were determined using a sodium MRF approach^49^. A relaxation time dictionary was created assuming a monoexponential T_1_ and biexponential ${{\rm{T}}}_{2}^{\ast }$ relaxation. Maximum absolute signal developments were simulated for a 20-pulse sequence for different sets of relaxation times with ranges as follows: T_1_ = [20:2:68] ms, ${{\rm{T}}}_{2l}^{\ast }$ = [14:2:60] ms, and ${{\rm{T}}}_{2s}^{\ast }$ = [1:1:13,14:3:60] ms (terms in brackets represent [min:step:max]). Measured mean signal evolutions for the different phantom compartments (from ROI measurements) over this 20-pulse FLORET sequence were compared to the simulations in the relaxation time dictionary. The simulated evolutions with the maximum correlation to the measured signal evolutions provided us with relaxation times for the three phantom compartment (see results in Table 2). Sequence settings were: flip angles *θ*_*i*_ = 43°, 25°, 99°, 114°, 23°, 122°, 43°, 72°, 68°, 24°, 36°, 56°, 27°, 16°, 107°, 26°, 32°, 79°, 45°, 36°, phases *ϕ*_*i*_ = 113°, 159°, 153°, 176°, 72°, 20°, 167°, 4°, 4°, 62°, 44°, 49°, 28°, 93°, 94°, 13°, 57°, 89°, 139°, 142°, and constant delays *τ*_*i*_ = 5 ms. The multipulse sequence used for the relaxation time determination was not the same multipulse sequence as used for the quantification results since we measured relaxation times earlier in this study. See Supplementary Information for figures: Figure S1 show an example of 20 axial images of the phantom, Figure S2 shows the ROIs used to measure the signal in the three compartments of the phantom, and Figure S3 shows a comparison of simulated and measured signal evolutions over 20 pulses used to calculate the phantom relaxation times. See Table 2 for the results.

### Sequence optimization

Sodium acquisitions were performed using a multipulse 3D UTE non-Cartesian FLORET sequence^39^ with N RF pulses, characterized by varying flip angles *θ*_*i*_, phases *ϕ*_*i*_ and constant delays *τ*_*i*_ after each pulses of index *i*, with *i* = 1 to N. Data was acquired during *τ*_*i*_, yielding N images. The multipulse sequence was optimized such that the Pearson correlation coefficient of the simulated maximum signal evolutions over N pulses was minimized between compartments 1 (IC) and 2 (EC). Since these two compartments of the model have different but close relaxation times, they were assumed to be the more difficult to decorrelate, while compartment 3 (CSF) has both monoexponential T_1_ and T_2_ and is easily decorrelated from the two other compartments.

To estimate the optimal set of parameters for the multipulse sequence in order to achieve minimal correlation between the signals from the compartments, the signal evolutions were simulated and optimized via randomization procedures. More specifically, the signal for the different compartments was simulated blockwise for three consecutive pulses with randomized phases and flip angles. Flip angles *θ*_*i*_ were chosen in the range [0°, 150°] and phases *ϕ*_*i*_ were chosen in the range [0°, 180°]. Minimizations were also performed including variable delays *τ*_*i*_, but they didn’t improve the results and therefore a fixed *τ*_*i*_ = 5 ms was included in all subsequent sequence optimizations. After several hundred iterations with random variations of *θ*_*i*_ and *ϕ*_*i*_, the group of first three pulses with minimized correlation between the signal evolutions from compartments 1 and 2 was determined. The following optimal group of three pulses was determined in the same way, while keeping the first three pulses fixed. The same procedure was applied for the next tree pulses, until we reached the number of pulses N. Pulse sequence settings obtained under this procedure were subsequently fine-tuned for *θ*_*i*_ and *ϕ*_*i*_ for further correlation minimization between the 15-point maximum signals of compartments 1 and 2 using the *fmincon* function in Matlab. With this procedure, a pulse sequence with N = 15 was optimized for maximum signal decorrelation between the three brain compartments using the relaxation times listed in Table 2.

**Figure 8:**
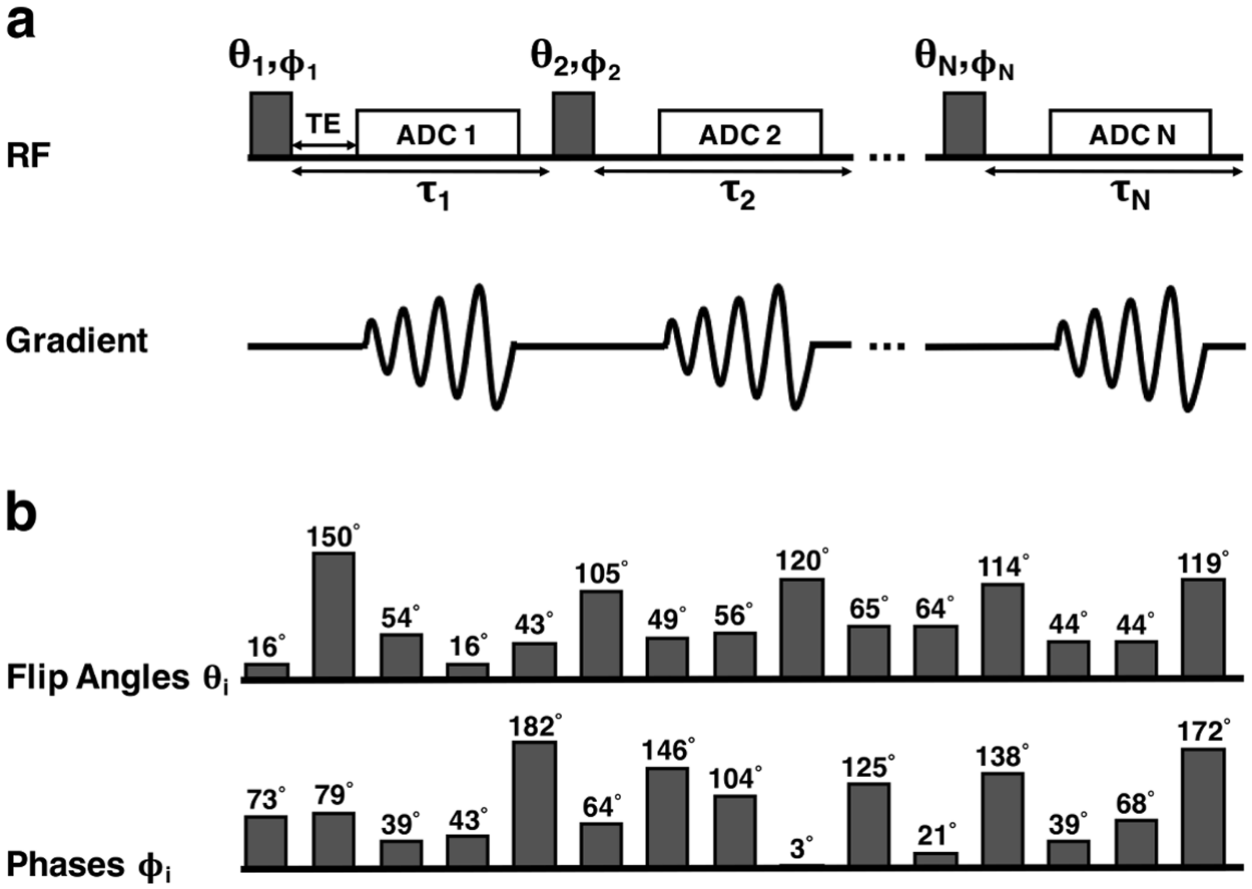
RF pulse sequence. (**a**) N-pulse FLORET sequence design with varying flip angles *θ*_*i*_, phases *ϕ*_*i*_, and time delays *τ*_*i*_ between the RF pulses (*i* = 1 to N). (**b**) Set of flip angles *θ*_*i*_ and phases *ϕ*_*i*_ optimized for the 15-pulse sequence as used in the present study in phantom and in brain. Delays *τ*_*i*_ were chosen to be constant, *τ*_*i*_ = 5 ms. See Methods for entire set of sequence parameters.

Sodium images were acquired using the same 15-pulse FLORET sequence in both phantom and brain (see Fig. 8). This sequence was originally optimized using brain relaxation times as described above. The RF parameters were as follows:

$$\begin{array}{rrllllllllllllllll}{\theta }_{i} & (^\circ ) & = & 16 & 150 & 54 & 16 & 43 & 105 & 49 & 56 & 120 & 65 & 64 & 114 & 44 & 44 & 119\\ {\phi }_{i} & (^\circ ) & = & 73 & 79 & 39 & 43 & 182 & 64 & 146 & 104 & 3 & 125 & 21 & 138 & 39 & 68 & 172\\ {\tau }_{i} & ({\rm{ms}}) & = & 5 & 5 & 5 & 5 & 5 & 5 & 5 & 5 & 5 & 5 & 5 & 5 & 5 & 5 & 5\end{array}$$

Simulation results were the absolute maximum signal evolutions for three different compartments during this 15-pulse sequence, and correspond to the entries of the *λ* matrix. For the quantification, signal evolutions were normalized to the maximum of the fluid signal development (either 0% agar or CSF, see calibration section). After the last pulse and last delay (*τ*_*n*_ = 5 ms), a longer delay assigned by the TR chosen for the acquisition was included in order to allow full relaxation of the sodium spins before the next 15-pulse acquisition (next interleaf in k-space for each of the 15 images).

### Proton MRI acquisitions

As a basis for brain masks, as well as for anatomical comparison, a ^1^H MRI acquisition with a magnetization prepared rapid acquisition gradient echo (MPRAGE) pulse sequence was performed with the following parameters: TR = 2300 ms, TE = 2.86 ms, resolution = 1.25 mm isotropic, acquisition time TA = 5 min, 1 average.

### Sodium MRI acquisitions

All sodium acquisitions were performed using the non-Cartesian FLORET sequence^18,39^.

- **Single-FLORET in brain:** TR = 150 ms, TE = 0.4 ms, 3 hubs at 45°, number of interleaves/hub = 103, resolution = 5 mm isotropic, TA = 8 min, number of averages = 10.
- **Multi-FLORET in phantom:** TR = 690 ms, TE = 0.4 ms, 3 hubs at 45°, number of interleaves/hub = 818, resolution = 3.6 mm isotropic, data acquisition (analog-to-digital converter) duration T _*ADC*_ = 4060 *μ*s, RF pulse duration = 1 ms, number of pulses = 15, *τ*_*i*_ = 5 ms, number of averages = 6, TA = 28 min/average. The 15 pulses (flip angles and phases) are listed in the Sequence optimization sub-section (see above) and in Fig. 8b.
- **Multi-FLORET in brain:** TR = 470 ms, TE = 0.4 ms, 3 hubs at 45°, number of interleaves/hub = 378, resolution = 5 mm isotropic, T _*ADC*_ = 4060 *μ*s, RF pulse duration = 1 ms, number of pulses = 15, *τ*_*i*_ = 5 ms, number of averages = 4 (except volunteer 4 who had only 2 averages), TA = 9 min/average. The 15 pulses (flip angles and phases) are listed in the Sequence optimization sub-section (see above) and in Fig. 8b.

The same sequence of RF pulse angles and phases was used in brain and in phantom. In all multipulse acquisitions, the gradients of the FLORET trajectory were all refocused at the end of each ADC acquisition following each RF pulse, in order to generate a fully refocused magnetization vector before application of the next RF pulse.

### SNR

We determined the SNR for each of the 15 sodium brain images individually. To measure the signal, we took the mean magnitude signal in a ROI over the whole brain in four consecutive slices for each of the 15 datasets. To assess the noise, we measured the whole magnitude signal in four consecutive noisy slices outside the brain, for all of the 15 datasets. SNR was calculated as the mean brain ROI values divided by standard deviation of the noise values.

### Data quantification processing

The final quantification to generate *α*_1_, *α*_2_, *α*_3_, and *C*_1_ maps after sodium data acquisition was processed in four steps:

1. **ROI in compartment 3 (0% gel or CSF):** After reconstruction of the 15 MR images from the multipulse sequence, we determined the signal evolution for compartment 3 (0% gel or CSF) out of the measured data. We measured the mean magnitude signal in a ROI over three consecutive slices in the CSF ventricles (for brain data quantification) or in the 0% agar gel (for phantom data quantification) after each of the 15 pulses, and normalized it to its maximum value over the 15 pulses. This measured normalized signal evolution for compartment 3 corresponds to its entries in the *λ* matrix, *λ*_*i*3_, with *i* = 1 to 15 (see equation 2). Moreover, the signal evolution of compartment 3 served as a basis for the calibration and correction steps.
2. **Calibration:** The ^23^Na concentration in compartment 3 (0% gel phantom or CSF brain compartment) was assumed to be constant with *C*_3_ = 140 mM. The signal evolution of compartment 3 (from ROI measurement, see above) served as reference for the calibration. All 15 images were divided by the signal evolution of compartment 3 over the 15 pulses, respectively. These normalized images were subsequently multiplied by the normalized entries *λ*_*i*3_ for compartment 3 and multiplied by 140 mM for the calibration.
3. **Correction Steps:** In order to improve the concordance between measured and simulated signal developments, and therefore to make the quantification more accurate, two correction steps were included in the data processing. A frequency offset dictionary was created simulating the 15-pulse signal developments for the three compartments with different frequency offsets in the range [−40, +40] Hz in steps of 2 Hz. At the same time, a ${B}_{1}^{+}$ correction was also included in the dictionary, according to the same principle simulating signal developments for linear flip angle corrections with ${B}_{1}^{+}$ factors ranging from [0.8, 1.2] in steps of 0.02, that were applied to all the flip angle values *θ*_*i*_. The values of *λ*_*i*3_ from the scan data served as reference. Comparing the *λ*_*i*3_ signal evolution to the signal evolutions from the complete corrected dictionary, with regard to maximized correlation, provided frequency offset and ${B}_{1}^{+}$ correction values. Including these optimal frequency offset and ${B}_{1}^{+}$ corrections, a new *λ* matrix for compartments 1 and 2 was re-simulated for subsequent data quantification. For compartment 3, we used *λ*_*i*3_ obtained from the measured data for the quantification.
4. **Quantification:** Sodium quantification was subsequently carried out by applying equations 5 voxelwise, yielding 3D maps of *α*_1_, *α*_2_, *α*_3_ and *C*_1_. The positive real values of these metrics were used to create the maps.

### Data Availability

The datasets generated during and/or analysed during the current study are available from the corresponding author on reasonable request.

## Electronic supplementary material

Supplementary Information
