# Supplementary material for: Multipulse sodium magnetic resonance imaging for multicompartment quantification: Proof-of-concept
Source: Sci Rep. 2017 Dec 12;7:17435. doi: 10.1038/s41598-017-17582-w (PMC5727256; doi:10.1038/s41598-017-17582-w)
Supplement: Supplementary file 1 — Supplementary Information [file 41598_2017_17582_MOESM1_ESM.pdf]

Supplementary Information

Multipulse sodium magnetic resonance imaging for multicompartment quantification: Proof-of-concept

Alina Gilles[1,2], **Armin M Nagel**[2], **and Guillaume Madelin**[1,*]

[1]Center for Biomedical Imaging, Department of Radiology, New York University School of Medicine, New York, NY 10016, USA
[2]Institute of Radiology, University Hospital Erlangen, Friedrich-Alexander-University Erlangen-Nuremberg, 91054 Erlangen, Germany
[*]Corresponding author: guillaume.madelin@nyumc.org

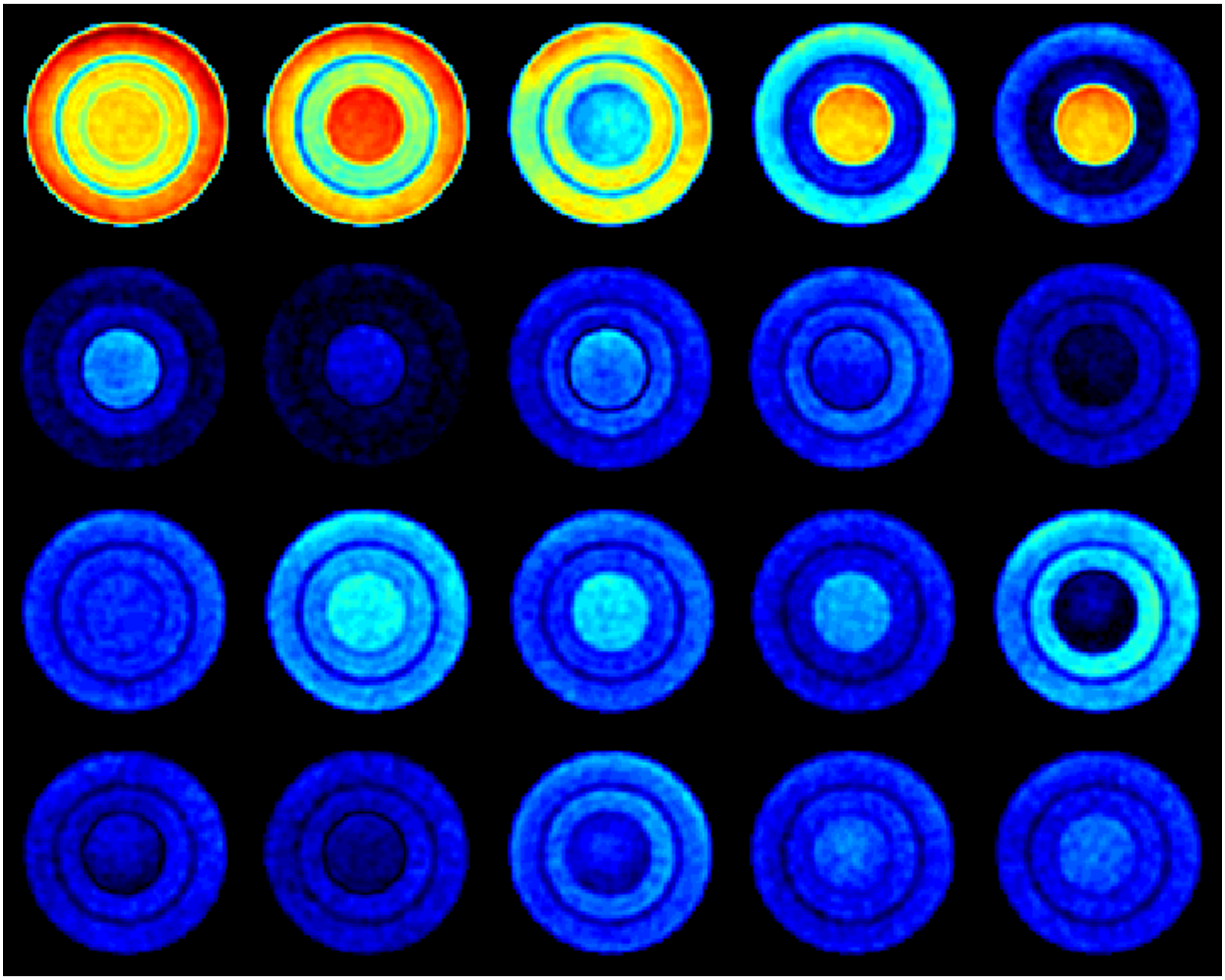

Figure S1. Acquisitions for relaxation times measurements in phantom. Sodium signal acquisitions of the three-compartment phantom over the 20-pulse FLORET sequence used for relaxation time measurements. The 20 RF pulses with flip angles were $\theta_i = 43^{\circ}$, 25°, 99°, 114°, 23°, 122°, 43°, 72°, 68°, 24°, 36°, 56°, 27°, 16°, 107°, 26°, 32°, 79°, 45°, 36°, with phases $\phi_i = 113^{\circ}$, 159°, 153°, 176°, 72°, 20°, 167°, 4°, 4°, 62°, 44°, 49°, 28°, 93°, 94°, 13°, 57°, 89°, 139°, 142°, and constant delays $\tau_i = 5$ ms between the pulses.

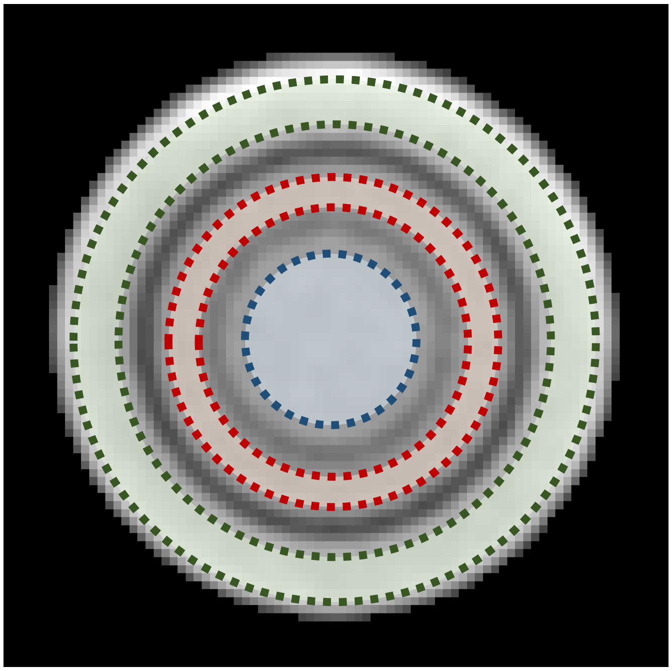

Figure S2. ROIs in phantom. Examples of the three ROIs (red: 8% agar gel, green: 4% agar gel, blue: 0% agar gel) in one central axial slice used to measure the absolute signal evolutions of the three different phantom compartments over a 20-pulse FLORET sequence.

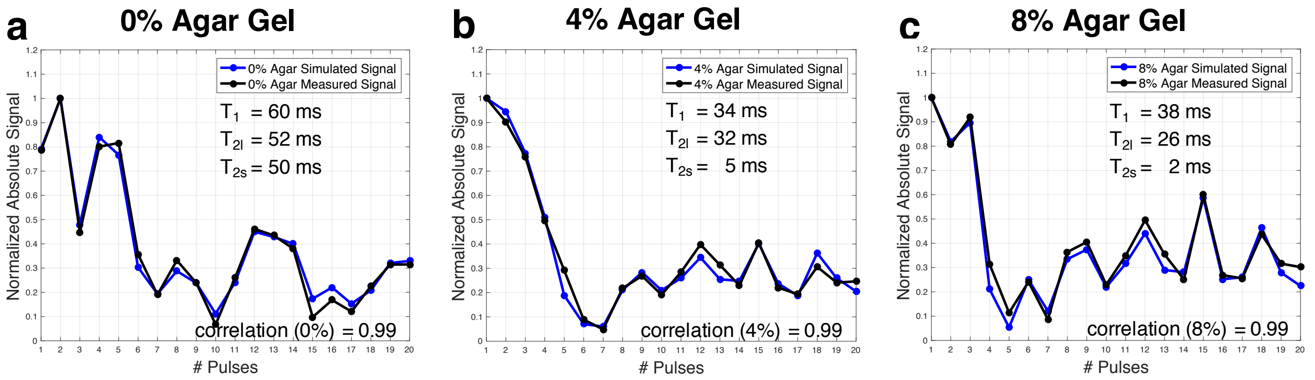

Figure S3. Measured signals for relaxation times matching. Measured normalized ROI ^{23}Na signal evolutions (black) over 20-pulse FLORET sequence for three different phantom compartments, a: 0% agar gel, b: 4% agar gel, c: 8% agar gel, and corresponding ^{23}Na normalized absolute signal simulations from relaxation time dictionary that show maximum correlation to the measured signals. The correlations between measured signal evolutions and matching relaxation time dictionary simulations are 0.99 for all three phantom compartments. The matching simulations provide relaxation times for the three phantom compartments: 0% agar gel: T_1 = 60 ms, T^*_{2l} = 52 ms, T^*_{2s} = 50 ms; 4% agar gel: T_1 = 54 ms, T^*_{2l} = 32 ms, T^*_{2s} = 5 ms; 8% agar gel: T_1 = 38 ms, T^*_{2l} = 26 ms, T^*_{2s} = 2 ms.
